# Genotyping-by-Sequencing in a Species Complex of Australian Hummock Grasses (*Triodia*): Methodological Insights and Phylogenetic Resolution

**DOI:** 10.1371/journal.pone.0171053

**Published:** 2017-01-30

**Authors:** Benjamin M. Anderson, Kevin R. Thiele, Siegfried L. Krauss, Matthew D. Barrett

**Affiliations:** 1 School of Plant Biology, The University of Western Australia, Crawley, Western Australia, Australia; 2 Kings Park and Botanic Garden, Botanic Gardens and Parks Authority, Kings Park, Western Australia, Australia; 3 Western Australian Herbarium, Department of Parks and Wildlife, Kensington, Western Australia, Australia; Saint Mary's University, CANADA

## Abstract

Next-generation sequencing is becoming increasingly accessible to researchers asking biosystematic questions, but current best practice in both choosing a specific approach and effectively analysing the resulting data set is still being explored. We present a case study for the use of genotyping-by-sequencing (GBS) to resolve relationships in a species complex of Australian arid and semi-arid grasses (*Triodia* R.Br.), highlighting our solutions to methodological challenges in the use of GBS data. We merged overlapping paired-end reads then optimised locus assembly in the program PyRAD to generate GBS data sets for phylogenetic and distance-based analyses. In addition to traditional concatenation analyses in RAxML, we also demonstrate the novel use of summary species tree analyses (taking gene trees as input) with GBS loci. We found that while species tree analyses were relatively robust to variation in PyRAD assembly parameters, our RAxML analyses resulted in well-supported but conflicting topologies under different assembly settings. Despite this conflict, multiple clades in the complex were consistently supported as distinct across analyses. Our GBS data assembly and analyses improve the resolution of taxa and phylogenetic relationships in the *Triodia basedowii* complex compared to our previous study based on Sanger sequencing of nuclear (ITS/ETS) and chloroplast (*rps16-trnK* spacer) markers. The genomic results also partly support previous evidence for hybridization between species in the complex. Our methodological insights for analysing GBS data will assist researchers using similar data to resolve phylogenetic relationships within species complexes.

## Introduction

Next-generation sequencing data sets are becoming increasingly accessible for addressing phylogenetic and biosystematic questions. Approaches to generating these data sets (reviewed in [1]) vary in their cost in terms of time and money, their requirement for existing genomic knowledge, their applicable evolutionary time (and hence taxonomic) scale, and the quality and sample coverage of the data. It is not always clear which approach will be most efficient and effective for addressing a given research question. In addition, analytical tools and approaches for resolving evolutionary relationships with genomic data are areas of ongoing research and testing [2–7], empirical application [8–12], and debate about best practice for phylogenetic inference [13,14].

Genotyping-by-sequencing (GBS [15]) is a next-generation sequencing approach based on restriction site-associated DNA (RAD) sequencing [16] and, like RAD sequencing, uses a restriction enzyme to generate thousands of loci as a reduced representation of a genome. The primary difference between the two methods is whether there is a random shearing step prior to amplification of fragments. In RAD sequencing, random shearing of fragments followed by adaptor ligation allows amplification of fragments from a single restriction site cut, while in GBS there is no shearing step, so only fragments with two close restriction sites are sequenced. The fewer steps and reduced sample handling as well as the simpler adaptor design make GBS more technically straightforward than RAD sequencing, with further cost reductions possible through reduced read depth [15]. As a result, GBS is relatively accessible for researchers lacking the equipment or protocols for custom library preparation and sequencing, and is available from commercial service providers. It can be used for non-model systems or in combination with a genomic reference (e.g. [17]). Refinements to the original method have been made by some studies (e.g. [17,18]), including normalising sample concentrations prior to pooling and adding size selection.

The assembly and analysis of GBS data pose bioinformatic challenges. GBS raw data (often Illumina HiSeq single-end 100 bp reads) have been commonly assembled using the UNEAK pipeline [19] in TASSEL [20], although some studies have used Stacks ([21], e.g. [22]) or PyRAD ([23], e.g. [24]) instead. Because GBS adaptors are not specific to which end of a DNA fragment they bind, there is the potential for read duplication, and bioinformatic assembly needs to account for this, particularly when using paired-end sequencing. Appropriate parameter values for assembly programs also need to be explored, with a recent approach [25] advocating an exploration that aims to maximize recovered loci while minimizing error rates and intrapopulation genetic distances. Once the data set has been assembled, analyses have typically used the called single nucleotide polymorphisms (SNPs) in clustering/assignment or summary statistics methods (e.g. [17,26,27]). Other studies have included a coalescent approach and/or concatenated entire loci for phylogenetic inference (e.g. [12,24,28]). A challenge at the analysis stage is handling the inherently high proportion of missing data [29] caused by low coverage sequencing [30] and allelic drop-out [31]. Relative to other restriction site approaches like RAD, GBS is likely to have more missing data due to often lower coverage sequencing and to increased allelic drop out, since for fragments to be shared they need to retain two restriction sites rather than just one. The optimal way to extract phylogenetic information from these data sets is not yet established. Newer summary species tree approaches (e.g. [6,32,33]), which take gene trees rather than sequence alignments as input, have not typically been applied to reduced representation data sets, likely because these approaches require a gene tree from each locus and the loci recovered from GBS and RAD sequencing tend to be short (e.g. [11]). In this study of a species complex, we explore the analysis of GBS data using summary species tree approaches (among others) and address some of the methodological challenges mentioned above.

The genus *Triodia* R.Br. (Poaceae: Chloridoideae) comprises perennial hummock grasses found only in Australia [34], typically in arid and semi-arid regions with < 350 mm mean annual precipitation and low-nutrient soils [35,36]. As dominant components of the vegetation across much of central Australia, these grasses are ecologically important as food and habitat for animals (e.g. [37–39]). The genus currently comprises 73 described species [34,40–45], with more awaiting description either from collections from relatively unexplored areas or from in-depth study of taxonomically challenging complexes and broadly applied names.

The *Triodia basedowii* E.Pritz. species complex comprises two formally recognised species (*T*. *basedowii* and *T*. *lanigera* Domin) and five informally recognised taxa (see Table 1). Anderson et al. [46] have recently shown that the complex is most diverse in the Pilbara region of Western Australia, where there is at least one additional unrecognised taxon (*T*. "shova") and multiple cases of putative hybridization between taxa. Morphological distinction of taxa can be difficult due to overlapping and variable character states and morphological plasticity, lack of readily available fertile material, and potential hybrid intermediates. Anderson et al. [46] found support for the distinction of most of the informally recognised taxa, but also cases of incongruence between nuclear and chloroplast markers and low support for relationships among some taxa in the complex. Putative taxa thought to be distinct based on morphology (*T*. "wcoast", *T*. "broad", *T*. "shovb") were not clearly supported by integrated data. Subsequent field work has identified two additional putative taxa (*T*. "Panna" and *T*. "nana") as part of the *T*. *basedowii* complex in Western Australia.

**Table 1:** Putative taxa in the *Triodia basedowii* species complex and their designation in this study.

Taxon	Designation in this study
*T*. *basedowii* E.Pritz.	*T*. *basedowii*
*T*. *lanigera* Domin	*T*. *lanigera*
*T*. sp. Little Sandy Desert (S. van Leeuwen 4935)	*T*. "LSandy"
*T*. sp. Shovelanna Hill (S. van Leeuwen 3835)	*T*. "Shov"
*T*. sp. Warrawagine (A.L. Payne PRP 1859)	*T*. "War"
*T*. sp. Peedamulla (A.A. Mitchell PRP1636)	*T*. "Peed"
*T*. sp. Pannawonica (B.M. Anderson & M.D. Barrett BMA 89)	*T*. "Panna"
*T*. "shova"	*T*. "shova"
*T*. "shovb"	*T*. "shovb"
*T*. "wcoast"	*T*. "wcoast"
*T*. "broad"	*T*. "broad"
*T*. "nana"	*T*. "nana"

Names of informally recognised taxa with "sp." and a geographic epithet follow the Australian convention for undescribed taxa (see [47]) and are on the Australian Plant Census, while putative taxa designated with " " were coined by Anderson et al. [46] or in this study.

In this paper, we present a case study for assembling and analysing GBS data to resolve relationships among closely related species, using the *Triodia basedowii* species complex as our study system. We demonstrate a number of methodological improvements for handling and analysing GBS data that should assist researchers using this accessible and affordable technique in natural systems. In particular, we incorporate paired-end read merger as an initial step, employ the program PyRAD (and optimise assembly parameters) to reduce duplication in the data set, and apply new summary species tree analyses in addition to concatenation, coalescent and clustering analyses. We evaluate the robustness of the phylogenetic analyses to assembly parameter choices and highlight the differences between results from concatenation *versus* summary species tree approaches. We use our GBS results to test the distinction of species in the *T*. *basedowii* complex recognized in Anderson et al. [46] and improve the resolution of relationships between them, with implications for the taxonomy of the group and future studies of its evolution.

## Materials and Methods

### Taxon Sampling

**Fig 1:**
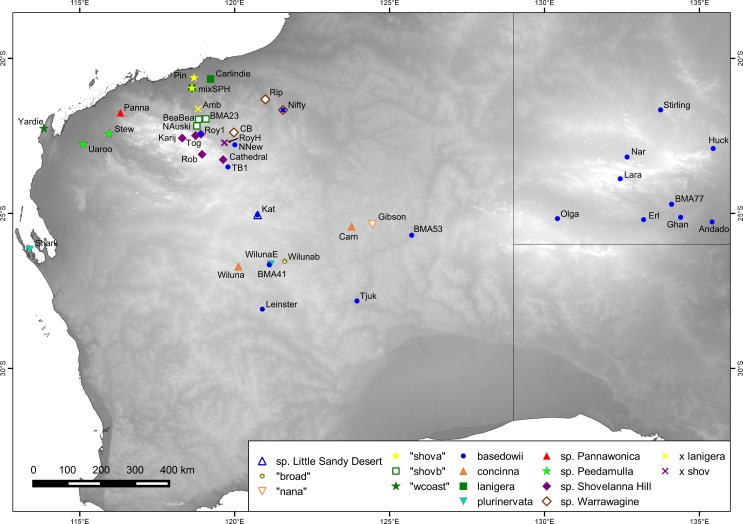
Sampling locations for populations from the *Triodia basedowii* complex and close relatives. The outgroups *T*. *concinna* and *T*. *plurinervata* are included. Elevation base map information from [48].

We sampled 139 plants from the *Triodia basedowii* complex for sequencing (Fig 1, S1 File), two of which were duplicated (i.e. comprised two DNA samples from the same plant). Sample identification was based on a previous study [46] and the recognition of distinct morphology in the field. Two species (*T*. *concinna* N.T.Burb. and *T*. *plurinervata* N.T.Burb., 16 plants from four populations) closely related to the complex were included as outgroups, while additional outgroups (*T*. *wiseana* C.A.Gardner and *T*. *intermedia* Cheel, 8 plants from two populations) were obtained from a separate study sequenced in the same GBS run.

### Ploidy Determination

As polyploidy is a common evolutionary process in angiosperms [49], it was important to survey ploidy variation in the complex to determine whether it needed to be accounted for in analyses. Ploidy was assessed for 28 populations (multiple samples) and 20 individuals (single samples per population) using flow cytometry. Flow cytometry has become the routine method of estimating plant ploidy variation ([50], e.g. [51]). Freshly collected leaf tissue was stored at 4°C until processing (up to 3 weeks). The flow cytometry protocol closely followed the propidium iodide protocol of Doležel et al. [50]. Nuclei were released from fresh plant tissue by finely chopping 1–4 cm of leaf tissue with a new double-edged razor blade in 1 ml of cold Tris.MgCl_2_-PVP buffer (200 mM Tris.HCl, 4 mM MgCl_2_, 0.5% V/V Triton X-100, adjusted to pH 7.5). After gently aspirating the buffer to suspend nuclei, 600 μl of the homogenate was filtered through 22 μm nylon mesh (product number 03-22/14, Plastok, Birkenhead, UK), and propidium iodide (Sigma-Aldrich, Castle Hill, NSW, Australia) and RNase (Qiagen Pty Ltd, Chadstone, VIC, Australia) added, giving a final concentration of 20 μg/ml and 20 μg/ml, respectively. Samples were screened on a Becton Dickinson FACScan flow cytometer, and the fluorescence intensity of a minimum of 6000 particles was recorded.

One sample per population was chopped with an internal size standard [cultivated plants from *Triodia wiseana* MDB 3889 (diploid, 2C = 2.64 ± 0.04 s.e.) or *T*. *wiseana* MDB 3981 (tetraploid, 2C = 4.97 ± 0.07 s.e.); errors propagated through reference calculations] to obtain estimates of holoploid genome size (see [52]). In most cases, these samples were prepared and measured three times to account for process and machine fluctuations, but one-off measurements are also included with notation to indicate this. *Solanum lycopersicum* L. 'Stupické polní rané' [50] was used as the reference standard (2C = 1.96 pg DNA) to establish the genome sizes of the *Triodia* reference plants, based on the average and standard deviation of five separate measurements. The remaining samples for each population were used to assess ploidy variation rather than measure genome size, so were chopped without a reference standard and the ploidy scored as diploid or tetraploid relative to an average fluorescence value of standards run separately. Observations with rarely-occurring and unusual ploidy levels were validated by repeating the measurement from new tissue.

### DNA Extraction, Library Preparation and Next-Generation Sequencing

Silica-dried leaf material was ground in liquid nitrogen and genomic DNA extracted using a CTAB method [53] as described in Anderson et al. [46] but with RNAse added prior to heating. DNA concentrations were quantified on a NanoDrop ND-1000 spectrophotometer and samples with concentrations between 139 ng/μl and 209 ng/μl sent to the Australian National University Biomolecular Resource Facility for library preparation and sequencing. Library preparation was modified from Elshire et al. [15], with primers and barcodes from Justin Borevitz [18]. Details of library preparation can be found in the supplementary material (S2 File). Briefly, 100–200 ng genomic DNA per sample was digested with the restriction enzyme PstI and custom adaptors, followed by ligation with T4 DNA ligase. Samples were purified with Ampure Beads and 80% ethanol washes, followed by individual PCR amplification and another purification step. Sample concentrations were assessed, and samples were pooled differentially to normalise concentrations. Pooled libraries were electrophoresed on an agarose gel and the 250–450 bp region cut out and purified for sequencing. Libraries were run across three lanes of an Illumina HiSeq 2000 with 96 samples per lane (including samples from a separate *Triodia* study), using 100 bp paired-end reads.

### Read Processing and Locus Assembly

**Fig 2:**
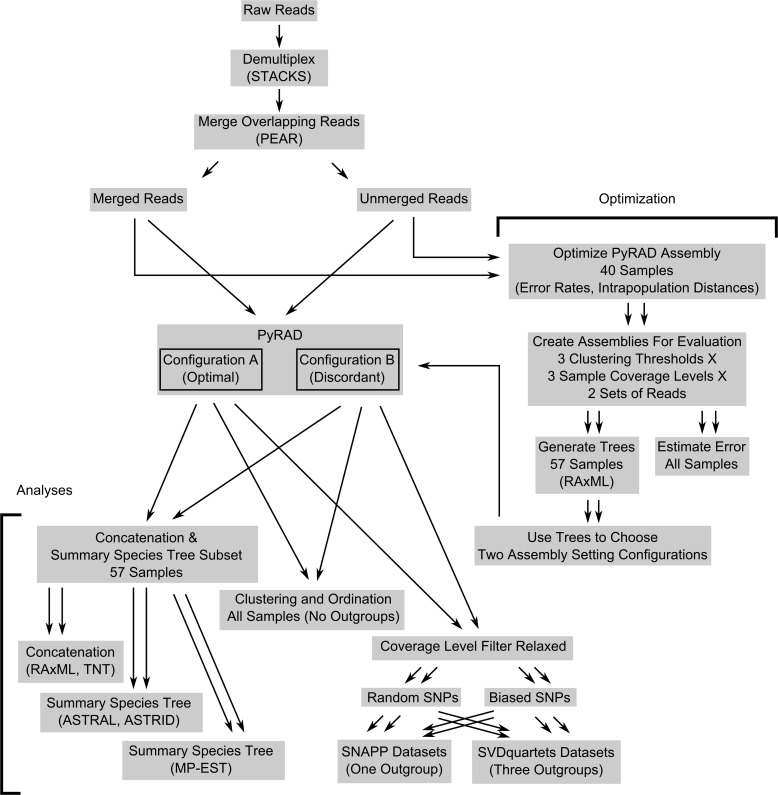
Schematic of bioinformatic and analytical steps presented in this study.

Raw paired-end reads from the ANU Biomolecular Resource Facility were demultiplexed with the sub-program *process_radtags* in Stacks v. 1.30 [21] using the options -q (discard low quality reads) and -c (remove reads with uncalled bases); this program was used because it could handle combinatorial barcodes. A schematic of the bioinformatic methods is provided in Fig 2 for clarity. To check for and merge overlapping reads, the demultiplexed reads were run through PEAR v. 0.9.8 [54] using default settings and a 16 bp minimum overlap.

Merged and unmerged reads were assembled separately into loci for calling SNPs using PyRAD v. 3.0.6 [23] with the "merged" and "pairgbs" data types respectively. PyRAD has the advantage over Stacks of allowing indel variation within loci and performing reverse complement clustering. Because the primers used in paired-end GBS do not bind specifically to each end of the DNA fragments, the first and second reads are interchangeable, so further bioinformatic processing (i.e. reverse complementing the second read to join it to the first) will duplicate loci. Reverse complement clustering addresses this issue. To determine appropriate assembly settings for PyRAD, we followed the approach of Mastretta-Yanes et al. [25] in using replicates and population samples to assess error rates and minimize genetic distances within populations. A subset of 40 samples, including two replicate pairs and ten populations (3–5 samples per population), was run through PyRAD for each value of six chosen parameters (Table 2), varying one parameter while keeping the other five at the default value.

**Table 2:** Parameters varied in PyRAD runs.

Parameter (designation)	Default value	Other values tested
#8 (d): minimum depth for a statistical base call	6	2, 3, 4, 8, 10
#9 (n): maximum number of low quality sites per read	4	2, 8
#10 (c): clustering threshold within and between samples	0.88	0.82, 0.85, 0.91, 0.94, 0.97
#13 (h): maximum number of individuals sharing a heterozygous site	3	5, 10
#25 (pl): ploidy filter = maximum number of alleles per locus	2	4, 8
#34 (mr): minimum number of reads for a dereplicate in an individual	1	2, 4, 8, 10

Parameter values were chosen to maximise the number of loci and SNPs recovered while minimising locus, allele and SNP error rates and genetic distances between samples from the same locality. Locus error is the number of loci missing in one replicate but not the other relative to the total loci; allele error is the number of shared loci differing in sequence between the replicates relative to the total shared loci; and SNP error is the number of SNPs differing between replicates relative to the total shared SNPs. Error rates and distances were calculated with custom scripts for R v. 3.2.1 [55] and the package APE v. 3.3 [56], following scripts from Mastretta-Yanes et al. [25]. A selection of scripts used in this study are included in the Supporting Information (S3 File).

After choosing parameter values for PyRAD, the full sample sets were assembled using those parameter values, and error rates calculated again using the two replicate sample pairs. In order to assess the impact on downstream phylogenetic inference of clustering threshold (a parameter that was observed to have a relatively large effect on the number of loci generated), we generated additional assemblies at thresholds 0.82 and 0.97. For each of the assemblies, we generated output at 25, 50, and 75% minimum sample coverage for loci to assess the impact of missing data.

### Concatenation Phylogenetic Analyses

A subset of 57 samples (4 per taxon where possible, 3 for *T*. "LSandy", 2 for *T*. "wcoast") were selected to give more even taxonomic coverage for phylogenetic inference. Unpartitioned PHYLIP files for the merged and unmerged data sets and combinations of clustering threshold and minimum sample coverage (2 data sets x 3 clustering thresholds x 3 minimum sample coverages = 18 alignments) were each analysed with RAxML v. 8.1.21 [57] using the GTRGAMMA model and a rapid bootstrap analysis [58] with 100 bootstrap replicates and maximum likelihood (ML) search in one run (option "-f a"). The resulting trees (S1 and S2 Figs) were visually compared for conflict, and two sets of assembly settings were chosen for further analysis. First, using the chosen clustering threshold (0.88/0.91), we selected coverage levels that produced congruent topologies for merged and unmerged data sets. Second, we selected clustering thresholds and coverage levels that produced a topology incongruent with the first. This was done to extract the most discordant phylogenomic signals observed when varying these parameters. The two assembly setting configurations (A: 0.88/0.91 at 50% minimum sample coverage, and B: 0.88/0.82 at 25% minimum sample coverage) were used to select input PHYLIP files, which were combined (merged + unmerged) for each assembly with a custom Python v. 2.7.2 [59] script and analysed in RAxML with the same settings as for the separate alignments. Trees from this analysis and subsequent analyses were visualised using FigTree v. 1.4.3pre [60].

Analyses on data sets with high proportions of missing data using RAxML may produce inflated support values [61], so we also analysed the concatenated alignments using a parsimony analysis in TNT v. 1.5beta [62]. PHYLIP files from the RAxML runs were converted to TNT format with a custom Python script. Gaps were treated as missing data rather than as a fifth character. Analyses used the "xmult" command to run 10 searches for the best tree from different starting points, implementing parsimony ratchet, drift and fuse, holding up to 100 trees for each search and keeping all found trees. The resulting tree(s) was then subjected to tree bisection-reconnection branch-swapping, and a strict consensus of the resulting trees generated. Branch support was computed with 100 bootstrap replicates, each replicate consisting of 10 searches holding up to 100 trees.

### Clustering and Ordination

SNPs in variant call format files for the merged and unmerged data assemblies using the chosen PyRAD parameter values (assembly setting configuration A; configuration B produced similar results and is not shown) were combined into a matrix using a custom Python script. The matrix was filtered to remove invariant and low coverage (< 50%) SNPs in R v. 3.2.1 [55]. The full SNP data set (multiple SNPs per locus) was used to calculate genetic distances between samples from the *T*. *basedowii* complex (no outgroups) using the dist.dna function in the R package APE v. 3.3 [56], with the default "K80" model and pairwise deletion of missing data. The resulting distance matrix was used to build a neighbour-joining tree (NJ tree; APE bionj function) and conduct principal coordinates analysis (PCoA; built-in cmdscale function) in R.

### Summary Species Tree Analyses

We applied summary species tree approaches (ASTRAL [6], ASTRID [32], MP-EST [33]), which take gene trees as input rather than DNA alignments and are able to handle large data sets. Our individual assembled GBS loci often had low coverage across samples and low information content for resolving relationships among samples, but the coalescent methods we used are able to handle polytomies in the input trees and to some degree missing taxa. We first created a PHYLIP file for each locus (same assemblies and samples as used in concatenation) from the PyRAD output for the merged and unmerged data using a custom Python script. For many of the loci, RAxML (run with the GTRGAMMA model) reported a warning that there was likely not a requirement for rate heterogeneity, so we ran jModelTest v. 2.1.7 [63,64] on the loci from the first chosen data set and found that 80% of loci fit a model without rate heterogeneity. To speed computation, we ran RAxML on each locus using the GTRCAT model without rate heterogeneity (-V), with 20 searches for the ML tree. For multi-locus bootstrapping (MLBS [65]), we generated the site resampling component by running 150 bootstrap replicates per locus in RAxML using the GTRCAT model without rate heterogeneity. These bootstrap trees were then sampled without replacement during 100 MLBS reps.

We used the sets of locus trees as input for ASTRAL v. 4.8.0 [6], which searches for a species tree that maximises a score reflecting the number of quartets induced by the tree that are present in the input tree set. ASTRAL was run using default settings and a name map file connecting samples to putative species. For MLBS, ASTRAL was run with the "-k bootstraps_norun" option to generate bootstrap input data sets without analysing them sequentially and the "-g" option to perform gene resampling. The resulting ASTRAL input data sets were executed multiple times in parallel to greatly speed computation. ASTRAL bootstrap trees were concatenated and summarised (using RAxML) on the tree from the first search, which was modified using a custom Python script to add branch lengths.

We also ran ASTRID v. 1.1 [32], which computes distances between taxa averaged over the input gene trees and creates a distance matrix, which is used with a distance-based method to construct a species tree. ASTRID was run with default settings and only the gene trees as input. For MLBS, we created a custom Python script to generate random sampling of loci with replacement for 100 replicates, coupled with selecting a RAxML bootstrap tree (without replacement) for each sampled locus to emulate site resampling. The resulting 100 sets of trees were used as input to ASTRID runs to generate a set of 100 ASTRID bootstrap trees. The bootstrap trees were concatenated and summarised (using RAxML) on the tree from the first search, again modified to add branch lengths.

We also analysed our locus trees using MP-EST v. 1.5 [33], which estimates branch lengths in coalescent units in addition to topology (ASTRAL and ASTRID only estimate topology). MP-EST uses the topologies of rooted input gene trees to calculate a pseudo-likelihood for the topology and branch lengths of the species tree based on the sets of rooted triplets in the species tree and the input gene trees. MP-EST assumes one lineage is sampled per species and thus does not estimate terminal branch lengths, and may perform poorly with non-random missing lineages in the input gene trees. To generate rooted trees (RAxML trees are unrooted), we used a custom R script and the package APE to examine each tree for an appropriate outgroup and root the tree on that outgroup if it was present or discard the tree if it was not. The preferred outgroup was any sample of *T*. *wiseana*, then *T*. *intermedia*, then either *T*. *plurinervata* or *T*. *concinna* if the other two were not present. MP-EST was executed with three runs for each of two configurations: 1) each sample was treated as a distinct lineage, and 2) groups of samples were assigned to species. A custom R script was used to extract and convert the resulting tree from the run with the highest likelihood. MP-EST creates trees with very long terminal branches to indicate they are not estimated, so a custom Python script was used to shorten terminals for visual clarity. For MLBS, the RAxML bootstrap trees were rooted as for the first run using a custom R script (or discarded if no outgroups were present), and the rooted trees used with the same custom Python script used to generate MLBS input tree sets for ASTRID. The resulting MLBS input tree sets were used for 100 replicates of three MP-EST runs for each of the two configurations, and the highest likelihood tree chosen from each set of the three runs as the MP-EST bootstrap tree for that replicate. The bootstrap trees were concatenated and summarised (using RAxML) on the best tree from the first run, again with branch lengths added.

### Unlinked SNP Species Tree Analyses

We applied a coalescent approach in SNAPP v. 1.2.2 [2] as implemented in BEAST v. 2.3.0 [66]. SNAPP takes unlinked biallelic SNPs as input rather than gene trees or alignments, but it also requires that SNPs be present in every species, which presents challenges when using GBS data (with large amounts of missing data). To maximise the chance of sampling a SNP for a species, we included up to seven samples per species where possible (fewer in some species due to limited sampling; see S1 File). Again, data sets were generated for the two clustering threshold combinations: 0.88/0.91 and 0.88/0.82 (coverage filter relaxed). After finding that unlinked SNPs sampled by PyRAD contained a high proportion of singletons, we also created an input set of SNPs using a custom Python script, biasing the SNP selection toward biallelic SNPs which had multiple occurrences of the rare allele. Custom R scripts were used to filter SNPs (biallelic, not invariant, present in at least one sample per taxon) and create an alignment Nexus file for BEAUti [66] in order to generate the xml file for SNAPP. We used default values for the priors on alpha and beta, calculated forward and reverse starting mutation rates, and set these to be sampled from the MCMC chain. SNAPP was executed with three parallel runs, each sampling every 400 generations from an MCMC chain, and was stopped manually when effective sample size (ESS) of the posterior was > 100 for most of the runs. ESS and convergence for the three runs was inspected with Tracer v. 1.6.0 [67] to confirm that runs had reached stationarity on the same likelihood value and that ESS for most parameters was well above 200 for the combined runs. We summarised a maximum clade credibility tree (with 10%, or more if the chain hadn't reached stationarity by that point, of trees discarded as burn-in) using mean heights in TreeAnnotator 2.3.0 (part of BEAST) and a cloudogram using DensiTree v. 2.2.2 [68].

We also used the unlinked SNPs as input to SVDquartets [69] as implemented in PAUP* v. 4.0a147 [70]. SVDquartets can handle missing taxa, so we broadened the sampling compared to SNAPP by including additional outgroups *T*. *plurinervata* and *T*. *wiseana*. The broader sampling and the tolerance for missing taxa resulted in a larger number of SNPs for the SVDquartets analyses compared to the SNAPP analyses. We again generated two unlinked SNP input data sets: a randomly selected SNP from each locus (PyRAD output), and biased selection toward biallelic SNPs with multiple occurrences of the rare allele. We ran two SVDquartets analyses per data set, one to estimate a species tree with multiple samples assigned to each species, and the other with all samples as individual lineages. Both analyses used default settings, randomly sampled 100,000 quartets, and included 100 bootstrap replicates. *Triodia wiseana* was used as the outgroup.

## Results

### Ploidy Determination

Samples from the *Triodia basedowii* complex and close relatives were found to be diploid or tetraploid (Table 3), in some cases with widely dispersed populations of tetraploids within otherwise diploid taxa. Ploidy is interpreted based on the distribution of DNA content within and between taxa. The lowest C-values observed within taxa are interpreted as diploid, while C-values approximately double the lower values are interpreted as tetraploid. The only taxon difficult to interpret in this way was *T*. "Panna", which comprised a single population sample and had the highest C-value (mean 5.15 pg). As its C-value is similar to that of tetraploids with diploid comparisons, it is interpreted as a tetraploid. Chromosome counts are necessary to confirm the interpreted ploidy for taxa in the complex.

**Table 3:** Ploidy measurements for the *T*. *basedowii* complex and close relatives.

Taxon	Population	Voucher	Mean 2C value(pg DNA)	Std. Error	Additional individuals assessed	Ploidy
*T*. *concinna*	Carn	BMA 45	4.11	0.07	-	tetraploid
*T*. *plurinervata*	Shark	Mayence s.n.	2.42	0.05	-	diploid
*T*. "ipluri"	WilunaE	BMA 42	4.08	0.06	-	tetraploid
*T*. *basedowii*	NNew	BMA 31	2.21	single	8 (diploid)	diploid
	Niftyb	BMA 37	1.97	single	-	diploid
	Tjuk	BMA 58	1.85	0.33	-	diploid
	-	BMA 64	4.58	0.07	-	tetraploid
	Huck	BMA 72	2.42	single	-	diploid
	-	BMA 73	4.77	single	-	tetraploid
	Nar	BMA 74	4.45	single	-	tetraploid
	Lara	BMA 75	2.22	single	-	diploid
	-	BMA 77	2.34	0.03	2 (diploid)	diploid
	Ghan	BMA 79	2.34	0.03	2 (diploid)	diploid
	Andado	BMA 80	4.84	single	-	tetraploid
	Erl	BMA 81	2.35	0.04	2 (diploid)	diploid
	Olga	BMA 83	2.36	0.06	2 (diploid)	diploid
	Roy1b	MDB 3932	2.15	single	4 (diploid)	diploid
	Wit	MDB 4127	2.29	0.04	9 (diploid)	diploid
	Stirling	MDB 4520	2.19	single	-	diploid
*T*. "broad"	Wilunab	BMA 43	2.35	0.04	-	diploid
*T*. "LSandy"	Kat	BMA 62	2.14	0.03	-	diploid
*T*. *lanigera*	Pea	BMA 13	4.09	0.07	9 (tetraploid)	tetraploid
	SPH	BMA 14	2.08	single	8 (diploid)	diploid
	mixSPH	BMA 16	2.08	0.03	9 (diploid)	diploid
	Carlindie	MDB 4099	2.34	single	9 (diploid)	diploid
*T*. "shovb"	NAuski	BMA 9, 10, 11	1.92	0.03	9 (diploid)	diploid
	BeaBea	MDB 4113	3.78	0.06	8 (tetraploid)	tetraploid
*T*. x *lanigera*	Amb	BMA 20	3.95	0.08	5 (tetraploid)	tetraploid
*T*. x *lanigera* shova	Amb	BMA 21	3.84	0.06	7 (tetraploid), 1 (diploid)	tetraploid
*T*. x shova	Amb	BMA 19	1.89	0.03	4 (diploid)	diploid
*T*. "shova"	mixSPH	BMA 15	1.97	0.05	8 (diploid)	diploid
	-	MDB 4437	1.87	double	-	diploid
*T*. "Peed"	Stew	MDB 3978	1.77	0.03	9 (diploid)	diploid
	Uaroo	MDB 4120	1.75	0.03	9 (diploid)	diploid
	-	MDB 4130	1.74	0.03	-	diploid
*T*. "wcoast"	Yardie	BMA 90	4.66	single	5 (tetraploid), 3 (diploid)	mixed
	-	BMA 91	4.30	0.08	4 (tetraploid)	tetraploid
*T*. "Panna"	Panna	BMA 89	5.15	0.11	9 (tetraploid)	tetraploid
*T*. "nana"	Gibson	BMA 49	2.13	0.03	-	diploid
*T*. "Shov"	-	BMA 6	2.16	double	-	diploid
	Roy1s	MDB 3933	1.95	single	9 (diploid)	diploid
	Karijini	MDB 4110	2.16	0.04	8 (diploid)	diploid
	-	MDB 4456	2.09	single	-	diploid
*T*. x shov war	RoyH2	MDB 4069, 70, 71	2.13	0.04	9 (diploid)	diploid
*T*. "War"	Niftyw	BMA 36	1.55	single	-	diploid
	CB	MDB 3944	1.84	single	7 (diploid)	diploid
	-	MDB 4073	1.73	single	4 (diploid)	diploid
	Rip	MDB 4082	1.72	0.03	7 (diploid)	diploid

Standard errors are based on three replicates of a single individual (not given when only one or two measurements were made).

Diploids were the dominant observed ploidy level, and the only ploidy level observed in *T*. *plurinervata*, *T*. "broad", *T*. "LSandy", *T*. "nana", *T*. "Peed", *T*. "shova", *T*. "Shov" and *T*. "War". *Triodia basedowii*, *T*. *lanigera*, and *T*. "shovb" had both diploid and tetraploid populations (exclusively so), while a mixture of diploids and tetraploids occurred in a population of *T*. "wcoast" and between morphological variants in the putatively hybrid population "Amb".

### Next-generation Sequencing

Sequencing generated 123–201 million paired-end reads per lane. Across two lanes, 21 samples from the *T*. *basedowii* complex failed to produce enough reads for further processing, leaving 120 samples for analysis. Of the 144 samples (120 *T*. *basedowii* complex samples plus 24 outgroup samples) that were successfully sequenced, there were 4.56 million reads per sample on average (1.28–8.06 million; two reads per fragment).

### Read Processing and Locus Assembly

Stacks demultiplexing and filtering retained on average 95.6% (93.1–97.1%) of reads per sample. PEAR merged on average 56.2% (44.6–68.4%) of demultiplexed reads, indicating size selection was not as effective as intended and that there was potentially a short fragment amplification bias. Statistics from PyRAD assembly showed that the merged data set had on average twice the read depth of the unmerged data, consistent with an amplification bias.

Choosing an optimal parameter value set was not straightforward due to the trade-offs between the number of loci/SNPs recovered, error rates (S3 Fig) and intra-population distances (S4–S10 Figs). Parameters with the greatest impact were clustering threshold (c) and minimum depth for a statistical base call (d). The chosen parameter values (Table 4) reflect a compromise for low but not the lowest error rates and intra-population distances, and an intermediate number of recovered loci/SNPs. Although polyploids are present in the data set, adjusting the ploidy filter (pl) only impacted the merged data set, possibly because the unmerged data set had insufficient read depth to recover additional alleles in polyploids. Because PyRAD does not output the additional alleles when the ploidy filter is relaxed (allowing more heterozygosity in loci instead), we chose to filter the merged data set to exclude loci with more than two recovered alleles per sample (pl = 2).

**Table 4:** Chosen parameters for PyRAD assembly of two data sets.

Parameter (designation)	Chosen merged	Chosen unmerged	Comment
#8 (d)	6	8	lower allele/SNP error; little effect on distances
#9 (n)	8	8	very little impact
#10 (c)	0.88	0.91	higher mean loci per sample, lower errors
#13 (h)	0.25 (10/40)	0.25 (10/40)	little impact
#25 (pl)	2	2	little impact (none for unmerged)
#34 (mr)	2	2	slightly lower error than mr = 1 (def)

Recovered loci/SNPs and error rates (Table 5) assessed using the two replicate samples (*T*. *basedowii* pop. Andado / *T*. "shova" pop. Pin) for the full data set illustrate the impact of clustering threshold and minimum taxon coverage. Higher allele error in the unmerged data probably reflects the longer loci (i.e. a longer sequence is more likely to have a sequencing error and can retain more differences when clustering based on a percentage similarity than a short sequence).

**Table 5:** Recovered loci/SNPs and error rates for various PyRAD assemblies of the full data sets.

Clustering threshold / read set	Min. taxon cover	Loci	SNPs	Locus error (bas / shova)	Allele error (bas / shova)	SNP error (bas / shova)
0.82 / merged	25%	3867	165,307	0.070 / 0.10	0.069 / 0.049	0.0045 / 0.0028
0.82 / unmerged	25%	5092	309,890	0.084 / 0.13	0.24 / 0.20	0.0048 / 0.0044
0.82 / merged	50%	1674	80,555	0.068 / 0.11	0.079 / 0.050	0.0053 / 0.0029
0.82 / unmerged	50%	2141	147,544	0.062 / 0.12	0.23 / 0.18	0.0049 / 0.0044
0.82 / merged	75%	478	21,568	0.050 / 0.052	0.066 / 0.038	0.0034 / 0.0022
0.82 / unmerged	75%	599	40,120	0.045 / 0.053	0.19 / 0.15	0.0052 / 0.0043
0.88 / merged	25%	3505	133,643	0.082 / 0.11	0.060 / 0.044	0.0035 / 0.0024
0.91 / unmerged	25%	3690	182,838	0.081 / 0.13	0.17 / 0.13	0.0023 / 0.0019
0.88 / merged	50%	1351	58,703	0.078 / 0.11	0.059 / 0.045	0.0033 / 0.0024
0.91 / unmerged	50%	1344	78,591	0.053 / 0.12	0.15 / 0.11	0.0021 / 0.0016
0.88 / merged	75%	386	15,481	0.067 / 0.049	0.044 / 0.027	0.0018 / 0.00078
0.91 / unmerged	75%	406	23,990	0.042 / 0.067	0.12 / 0.072	0.0015 / 0.00062
0.97 / merged	25%	1271	24,284	0.15 / 0.11	0.033 / 0.037	0.0024 / 0.0031
0.97 / unmerged	25%	722	18,347	0.11 / 0.13	0.096 / 0.095	0.0028 / 0.0037
0.97 / merged	50%	287	7013	0.20 / 0.14	0.0062 / 0.025	0.00052 / 0.0011
0.97 / unmerged	50%	92	3423	0.14 / 0.21	0.077 / 0.083	0.0017 / 0.0013
0.97 / merged	75%	74	1907	0.18 / 0.095	0.020 / 0.015	0.0016 / 0.00062
0.97 / unmerged	75%	22	889	0.045 / 0.091	0 / 0	0 / 0

### Concatenation Phylogenetic Analyses

**Fig 3:**
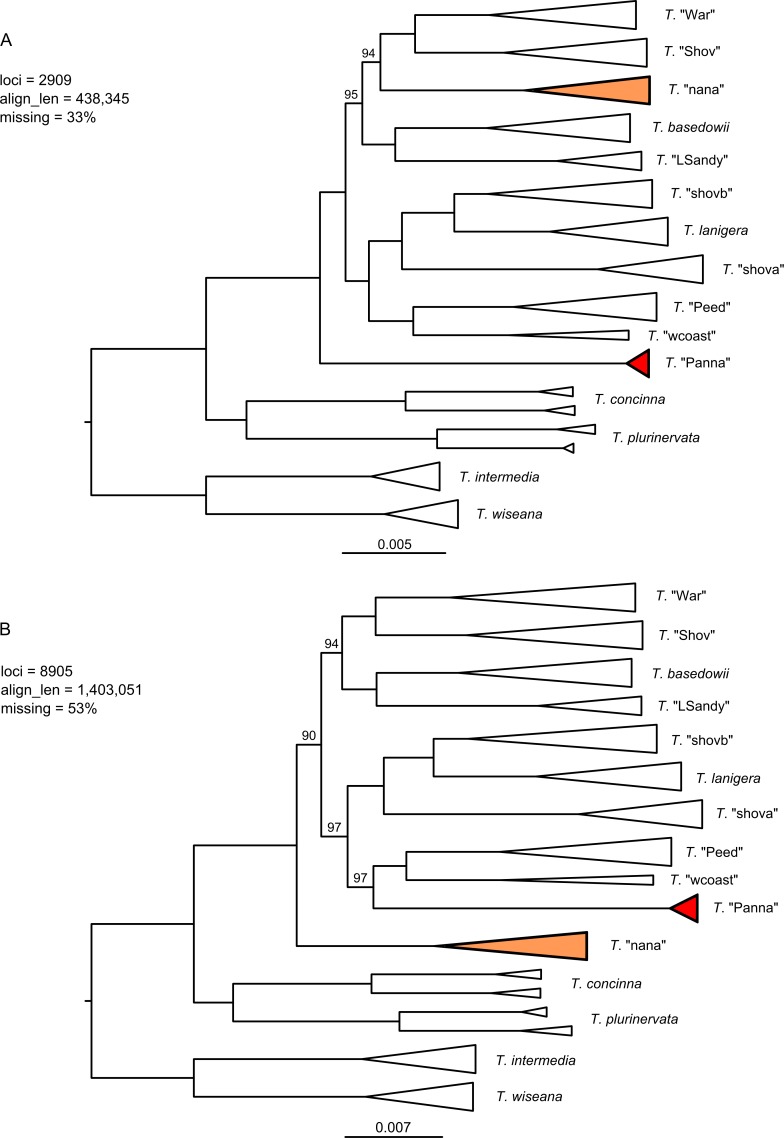
RAxML trees for the combined data set and two assemblies. A) 0.88/0.91 (merged/unmerged) clustering threshold, 50% minimum taxon coverage; B) 0.88/0.82 clustering threshold, 25% minimum taxon coverage. Support values from 100 bootstrap replicates are only shown for branches with <100% support. Scale bar units are RAxML branch lengths.

**Fig 4:**
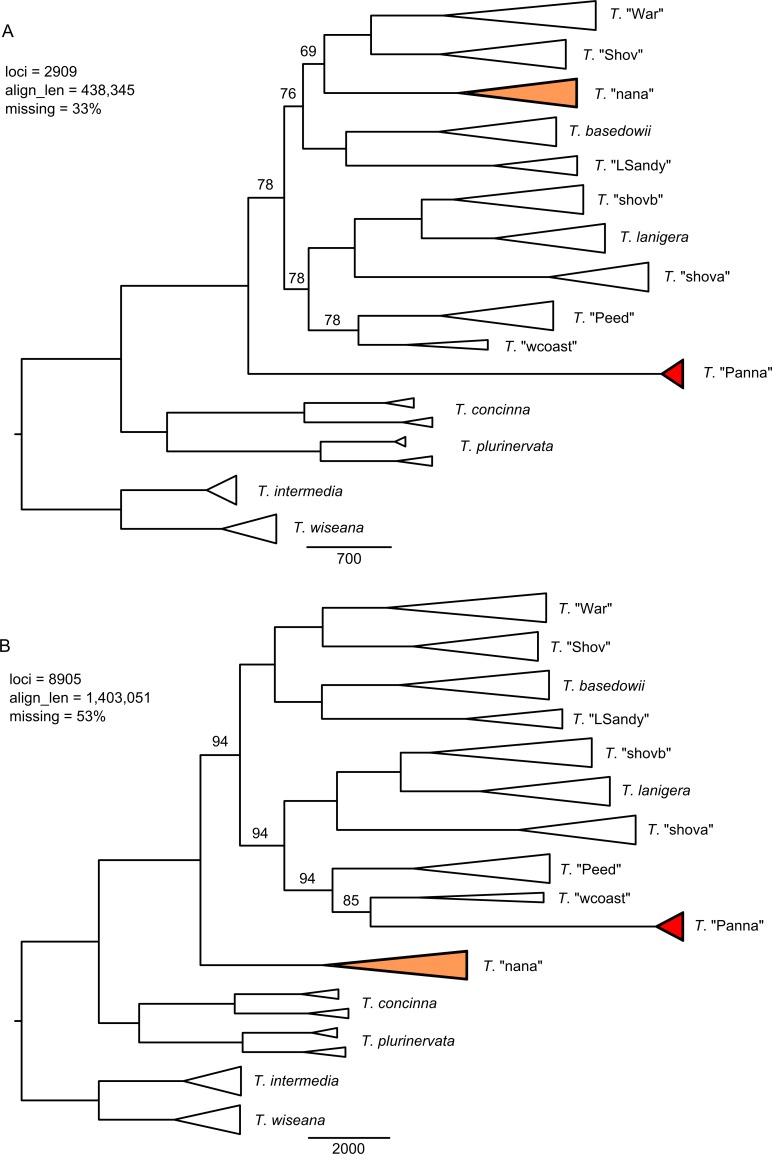
TNT maximum parsimony trees for the combined data set and two assemblies. A) 0.88/0.91 (merged/unmerged) clustering threshold, 50% minimum taxon coverage; B) 0.88/0.82 clustering threshold, 25% minimum taxon coverage. Support values from 100 bootstrap replicates are only shown for branches with <100% support. Scale bar units are differences.

The three clustering thesholds and three minimum sample coverage levels had substantial impact on the size of the resulting data sets and the resolution and topologies of RAxML trees for the merged and umerged data (S1 and S2 Figs). A higher clustering threshold tended to decrease the number of recovered loci; this was exacerbated by increasing the minimum sample coverage, as more variable loci were split into multiple loci that were then not present in enough taxa to pass the coverage filter. Within each clustering threshold, resolution of the RAxML trees improved with more loci and more missing data. RAxML analyses of the two chosen assembly setting configurations (A and B) with combined loci (merged + unmerged) produced well-supported and conflicting topologies (Fig 3). The topological conflict results from the placement of two taxa, *T*. "nana" and *T*. "Panna" (shaded in the trees). Other groups in the trees remain well-supported between the two data sets, such as the sister relationship between *T*. "Shov" and *T*. "War" (100%), which was not recovered previously [46]. A close relationship between *T*. *lanigera* and *T*. "shovb", with *T*. "shova" sister, is also consistently recovered with strong support (100%). Corresponding TNT maximum parsimony trees (Fig 4) support the same topologies as the RAxML trees, though with lower support for some clades for assembly configuration A and similar support to RAxML for configuration B.

### Clustering and Ordination

**Fig 5:**
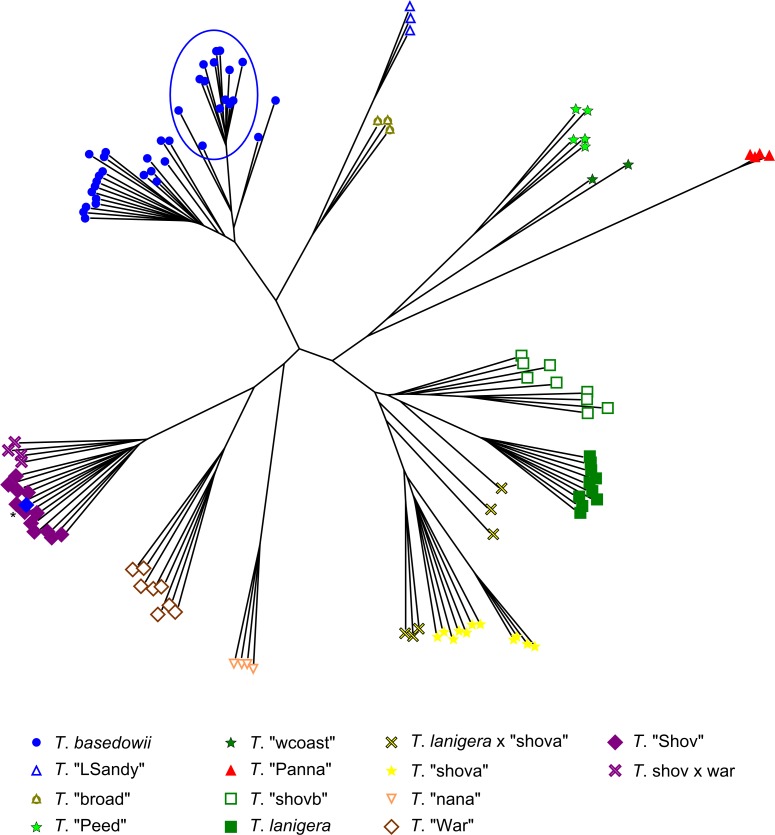
Neighbour-joining tree for samples from the *Triodia basedowii* complex. Distances are based on 119,449 SNPs, after filtering for 50% minimum taxon coverage and removing invariant SNPs. *T*. *basedowii* populations from central Australia are circled. The asterisked sample (blue diamond) was likely mislabelled as *T*. *basedowii* in the field (co-occurring with *T*. "Shov") and is probably a *T*. "Shov" sample.

**Fig 6:**
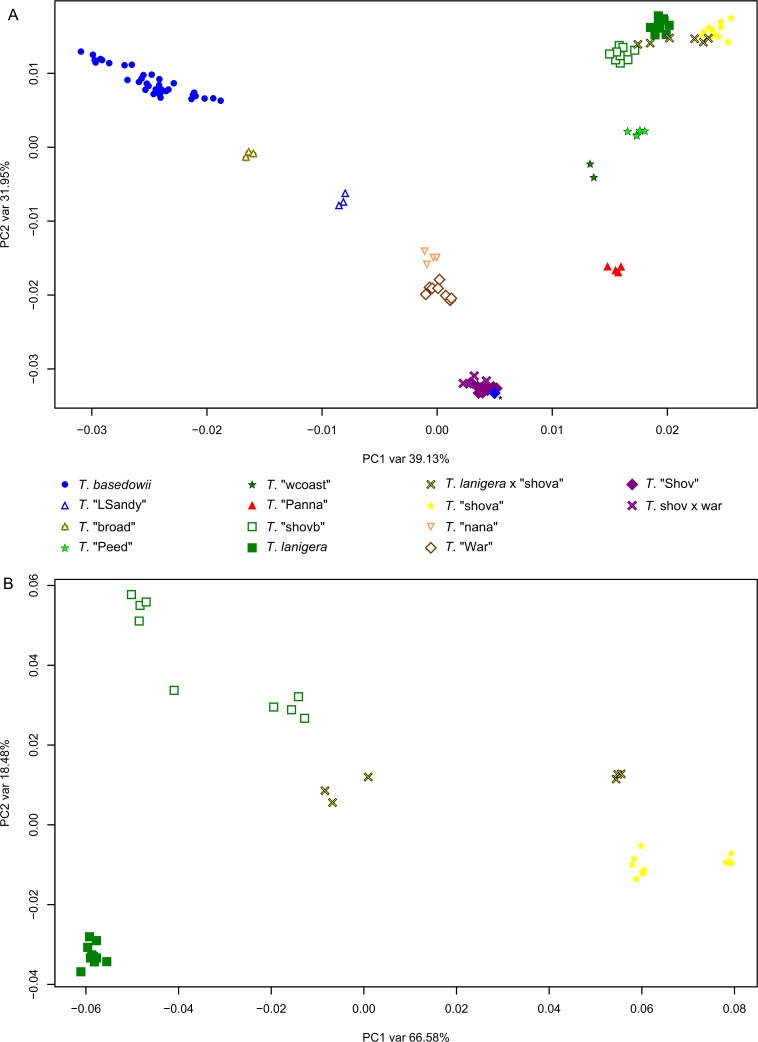
Principal coordinates analysis of samples from the *Triodia basedowii* complex. A) all samples, using 119,449 SNPs following filtering for 50% minimum taxon coverage and removing invariant SNPs; B) samples of *T*. *lanigera*, *T*. "shovb", *T*. "shova" and putative hybrids between them, using 44,365 SNPs after filtering as in A. The asterisked sample is likely mislabelled as *T*. *basedowii* (co-occurring).

After filtering, 119,449 SNPs were retained from 118 samples (excluding the two duplicates) within the *T*. *basedowii* complex (no outgroups). Both the NJ tree (Fig 5) and PCoA (Fig 6A) clearly show distinct clusters of samples with strong correspondence to *a priori* taxon identifications. The geographically widespread *T*. *basedowii* forms a distinct cluster. *T*. "LSandy" is distant from *T*. *basedowii*, with the *T*. "broad" samples clustering between them. *T*. "Shov" and *T*. "War" are clearly separated, with putative hybrids between them clustering with *T*. "Shov" rather than between the two taxa as might be expected. Samples from a population of mixed *T*. *basedowii* and *T*. "Shov" group distinctly with other samples from those species as they were identified in the field (with one exception shown by an asterisk). *T*. "Peed" and *T*. "wcoast" cluster closely but with some distinction, whereas *T*. "Panna" is quite distinct. *T*. *lanigera* groups closely with *T*. "shovb" and *T*. "shova" and their putative hybrids, which cluster between the parental taxa as expected (shown in greater detail in the PCoA in Fig 6B, based on a subset of 36 samples and 44,365 SNPs after filtering).

### Summary Species Tree Analyses

**Fig 7:**
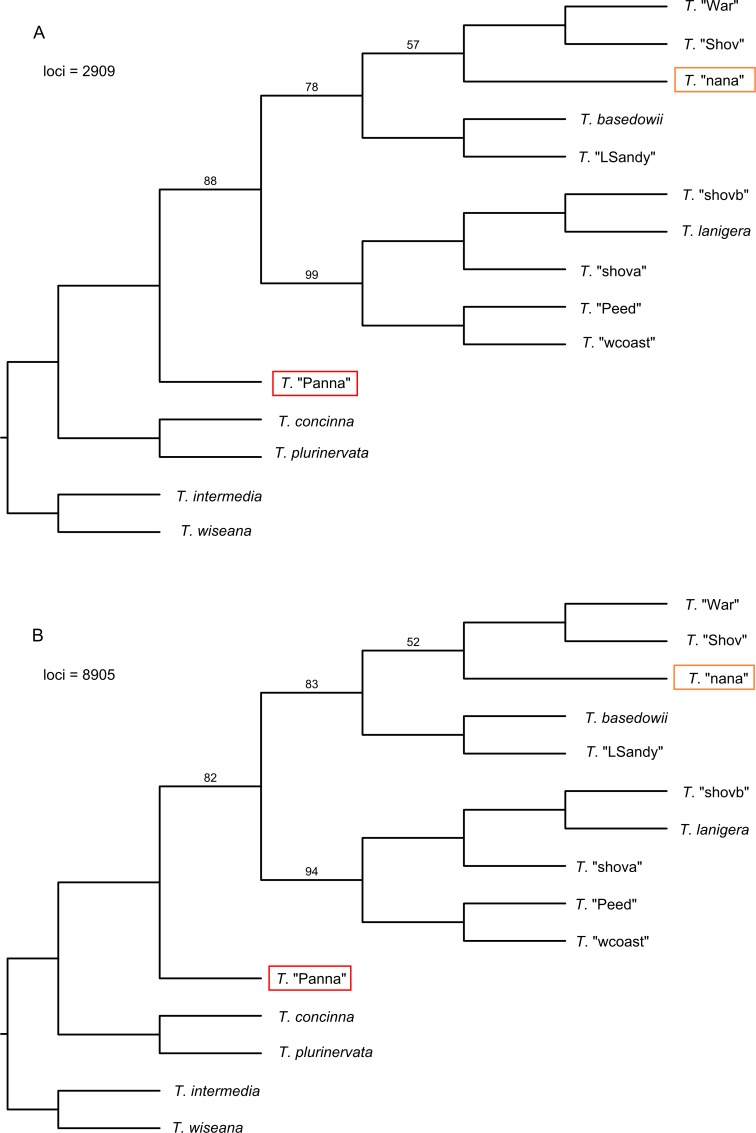
ASTRAL trees for the combined data set and two assemblies. A) 0.88/0.91 (merged/unmerged) clustering threshold, 50% minimum taxon coverage; B) 0.88/0.82 clustering threshold, 25% minimum taxon coverage. Support values from 100 multi-locus bootstrap replicates are only shown for branches with <100% support. Branch lengths are meaningless.

The ASTRAL analyses of data sets from configurations A and B produced trees (Fig 7) with the same topology but different levels of bootstrap support. The recovered topology is the same as the concatenated RAxML/TNT analyses of the data set for configuration A. Both ASTRAL trees have 100% support for clades supported in the concatenated analyses: *T*. "Shov" sister to *T*. "War", and the clade comprising *T*. *lanigera* and *T*. "shovb" with *T*. "shova" sister to them, among others. Low bootstrap support is associated with the position of *T*. "nana", and to a lesser degree with the sister relationship of *T*. "Panna" to the rest of the complex.

**Fig 8:**
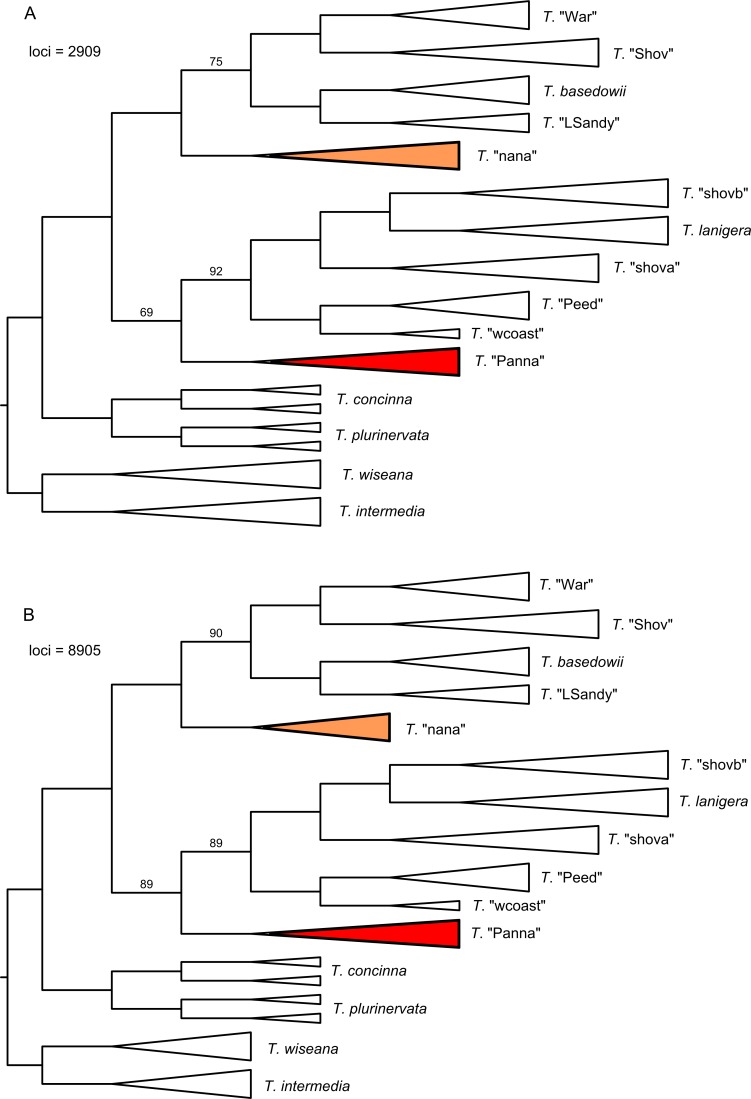
ASTRID trees for the combined data set and two assemblies. A) 0.88/0.91 (merged/unmerged) clustering threshold, 50% minimum taxon coverage; B) 0.88/0.82 clustering threshold, 25% minimum taxon coverage. Support values from 100 multi-locus bootstrap replicates are only shown for branches with <100% support. Branch lengths are meaningless.

As with the ASTRAL analyses, the ASTRID analyses of both data sets produced trees (Fig 8) with the same topology and differing bootstrap support values. The topology is incongruent, however, with the concatenation analysis of the data set for configuration A and the ASTRAL trees, having a different placement for *T*. "nana" (now sister to the clade containing *T*. "War", *T*. "Shov", *T*. *basedowii* and *T*. "LSandy") and *T*. "Panna" (now sister to the clade containing *T*. *lanigera*, *T*. "shovb", *T*. "shova", *T*. "Peed" and *T*. "wcoast"). These shifted positions are poorly supported for the data set based on configuration A (75% support for the clade sister to *T*. "nana"; 69% support for the clade containing *T*. "Panna"), but have good support for the data set based on configuration B (90% and 89%, respectively). The lower support for the clade sister to *T*. "nana" for the first data set reflects the tendency in some bootstrap replicates for *T*. "nana" to be recovered sister to *T*. "War" and *T*. "Shov" (as in the ASTRAL and concatenation trees).

**Fig 9:**
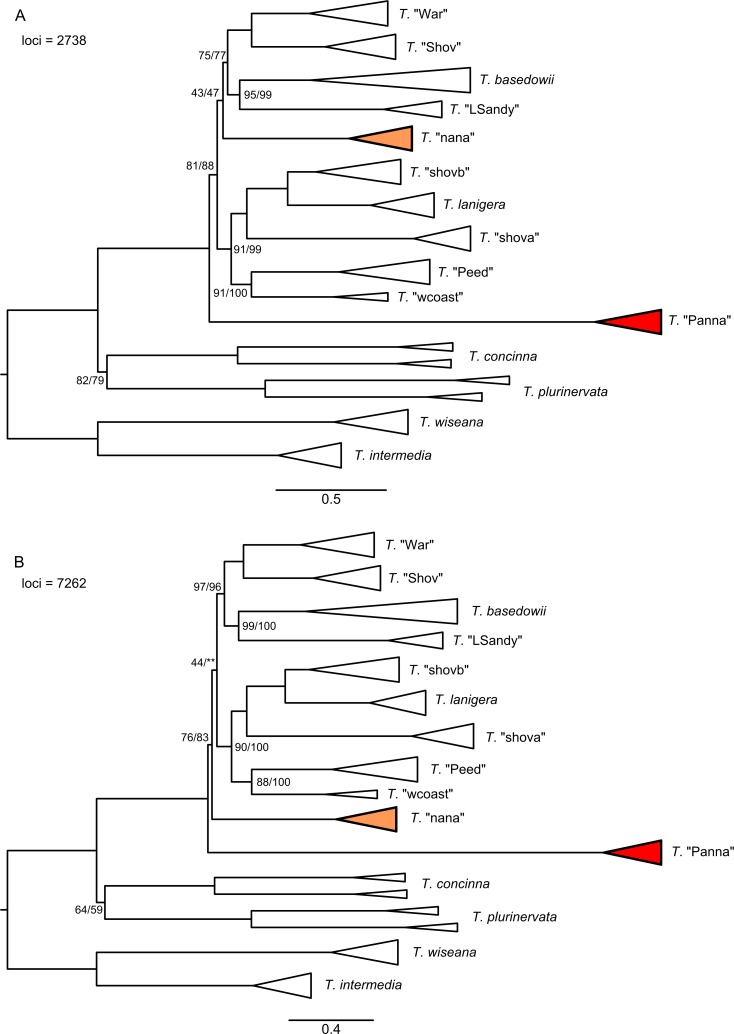
MP-EST trees (samples as species) for the combined data set and two assemblies. A) 0.88/0.91 (merged/unmerged) clustering threshold, 50% minimum taxon coverage; B) 0.88/0.82 clustering threshold, 25% minimum taxon coverage. Scale bars are in coalescent units. Terminal branch lengths are not estimated, so the length of collapsed groups (triangles) is not reliable. All shown branch lengths are estimated by MP-EST. Support values from 100 multi-locus bootstrap replicates are only shown for branches with <100% support, for both analyses (samples as species / groups of samples as species). **In the analysis with groups of samples as species, *T*. "nana" grouped with the top four taxa (as in A) with 46% support.

The requirement for rooted gene trees for MP-EST resulted in the exclusion of 171 loci from the data set based on configuration A and 1643 loci from the data set for configuration B. MP-EST analyses of both data sets resulted in largely congruent trees (Fig 9), the primary difference being the placement of *T*. "nana", although with < 50% bootstrap support. Otherwise, the topology is the same as was found in the ASTRAL analyses and the concatenation analyses of the data set for configuration A. Again there was strong support (100%) for the sister relationship between *T*. "Shov" and *T*. "War", and for the clade *T*. *lanigera* + *T*. "shovb" sister to *T*. "shova". There is marginal support (81% data set for configuration A, 76% data set for configuration B) for the sister relationship of *T*. "Panna" to the rest of the complex. Topologies and bootstrap supports were highly similar between analyses using individual samples as distinct lineages (trees in Fig 9) and using groups of samples as species, the only difference in topology being the placement of *T*. "nana" for the configuration B data set (with < 50% bootstrap support). Using individual samples as species allowed MP-EST to estimate branch lengths subtending each species, whereas analysing the data with samples assigned as groups to species allowed only the estimation of branch lengths subtending two or more species.

### Unlinked SNP Species Tree Analyses

#### SNAPP

After filtering the randomly sampled SNPs, 983 (72% singletons) and 1335 (67% singletons) unlinked biallelic SNPs shared across species (67 samples) were retained for SNAPP for clustering thresholds 0.88/0.91 and 0.88/0.82 respectively. The SNAPP analyses resulted in highly unresolved trees (S11 Fig), the topologies representing less than 0.2% and 1% of the 95% HPD sets for the two clustering threshold combinations. The cloudograms (S11 Fig) show the massive phylogenetic uncertainty. Only the sister relationship of *T*. *concinna* to the complex and the clade *T*. *lanigera* + *T*. "shovb" are supported (PP = 1.0) in the 0.88/0.91 tree, while the 0.88/0.82 tree also has support for the sister relationship of *T*. "shova" and *T*. *lanigera* + *T*. "shovb".

After filtering the biased SNPs, 986 (59% singletons) and 1293 (60% singletons) unlinked biallelic SNPs shared across species (67 samples) were retained for SNAPP for clustering thresholds 0.88/0.91 and 0.88/0.82 respectively. The resolution of the SNAPP trees (see S12 Fig) improved over the randomly selected SNP trees, but were again largely unresolved. The 0.88/0.91 tree (9.8% of the 95% HPD) has support (PP = 1.0) for the clade of *T*. *lanigera*, *T*. "shovb" and *T*. "shova" but without support for the sister relationship between *T*. *lanigera* and *T*. "shovb". There is support (PP = 0.99) for the sister relationship of *T*. "Shov" and *T*. "War" and marginal support (PP = 0.97) for the sister relationship of *T*. *basedowii* and *T*. "LSandy". The 0.88/0.82 tree (8.5% of the 95% HPD) similarly has support (PP = 1.0) for the clade of *T*. *lanigera*, *T*. "shovb" and *T*. "shova" with very low support (PP = 0.8) for the sister relationship between *T*. *lanigera* and *T*. "shovb".

#### SVDquartets

After filtering the randomly sampled SNPs, 14,411 (65% singletons) and 15,120 (62% singletons) unlinked SNPs from 78 samples were retained for SVDquartets for clustering thresholds 0.88/0.91 and 0.88/0.82 respectively. The species and lineage trees (S13 Fig) are partly resolved along the backbone of the complex, and there is support for some clades, but support values are not consistent between the species and lineage trees. For example, there is 100% bootstrap support for the sister relationship between *T*. "War" and *T*. "Shov" in the 0.88/0.91 species tree, but only 76% in the lineage tree. There is stronger and more consistent support for both the above relationship and the clade of *T*. *lanigera* + *T*. "shovb" (99/95%) sister to *T*. "shova" (100/90%) in the 0.88/0.82 species and lineage trees, respectively. While *T*. "Peed" was assigned samples of *T*. "wcoast" in species tree analyses, the *T*. "wcoast" samples sometimes grouped separately from other *T*. "Peed" samples in the lineage analyses, though without strong support.

After filtering the biased SNPs, 15,268 (52% singletons) and 16,001 (50% singletons) unlinked SNPs from 78 samples were retained for SVDquartets for clustering thresholds 0.88/0.91 and 0.88/0.82 respectively. The species and lineage trees (S14 Fig) are again only partly resolved, with slightly more resolution along the backbone for the 0.88/0.82 trees compared to the randomly sampled SNP analysis. There is strong support (98/98% and 96/91%) for the sister relationship of *T*. *basedowii* and *T*. "LSandy" for both clustering thresholds, in contrast to the randomly sampled SNP analysis which did not recover support for this relationship. There is again support for the clade of *T*. *lanigera* + *T*. "shovb" (100/95% and 100/100%) sister to *T*. "shova" (100/98% and 97/79%), but only marginal support (88/88% and <75/80%) for the sister relationship of *T*. "War" and *T*. "Shov".

## Discussion

Our approach to assembling and analysing GBS data highlights common bioinformatic challenges and reveals multiple ways to extract biologically meaningful signal from these data sets, especially in non-model systems. Our results also have implications for the biosystematics of the *Triodia basedowii* complex, with support for the recognition of multiple new species and greater resolution of relationships between species than was obtained in a previous study [46] based on Sanger sequencing of a few loci.

### Methodological Insights for Using GBS in a Species Complex

Using GBS to address phylogenomic questions presents methodological challenges that have implications for downstream analyses. While each study will face different obstacles, depending especially on the biology of the study system, we provide some common potential challenges and solutions that may be useful for researchers working with GBS data.

#### Sample targeting and taxon selection

When using GBS data in a species complex, preliminary sample targeting is important, as is getting a sense of what the data indicate might be taxa for more detailed analysis. While sequencing technologies are likely to continue to improve to the point that it is cost efficient and feasible to sequence massive numbers of samples from a given study system, we are not there yet, and researchers, especially those from labs with limited funding, have to carefully target samples for a GBS study. If the study system is poorly known, effectively targeting lineages is problematic, so knowledge of the system becomes vital for generating meaningful data, underscoring the importance of natural history collections and field-based observations. We were able to efficiently target taxa in the *T*. *basedowii* complex for sequencing only because of previous work (e.g. [46]) using herbarium specimens and extensive field observations and collections. For detecting potential genomic entities, we found that computationally quick distance-based analyses and even RAxML runs on a subset of the data provided relative genomic divergence and a guide for whether taxa were clustering distinctly. Some of the species tree methods we used (e.g. ASTRAL, SVDquartets, SNAPP) rely on the researcher to specify the species membership of samples, and we recommend relatively simple and fast clustering analyses for this *a priori* sample assignment process.

#### Paired-end sequencing and locus duplication

One of the advantages to GBS is the technical simplicity of adaptor design, but this presents a challenge for bioinformatic assembly. Because GBS libraries are constructed with adaptors that are not specific to which end of the digested genomic DNA fragments they bind, sequencing of very short fragments or, particularly, paired-end sequencing will lead to the duplication of loci in the data set. This duplication becomes less of an issue with single-end GBS data with effective size selection, but the benefits of added read length (increased information content per locus, which is important for phylogenomic analyses) from paired-end sequencing is lost. We addressed this duplication by using PyRAD, which, unlike UNEAK or Stacks, performs reverse complementation clustering during assembly and merges loci that had been constructed from fragments sequenced starting from opposite ends. A second source of duplication specific to paired-end sequencing occurs when there is poor size selection and small fragments are sequenced, resulting in read overlap. We observed this in our data set, where a large portion of our raw reads (56% on average) overlapped. Read overlap does not seem to be commonly addressed in GBS studies, but it also has the potential to lead to SNP duplication if reads overlap substantially. We addressed this overlap by merging those reads in PEAR prior to assembly with PyRAD.

#### Read depth, polyploidy and missing data

A challenge for using GBS is the large amount of missing data partly due to low coverage sequencing. In our study, this low coverage was more evident in the longer loci from unmerged reads, which had roughly half the read depth compared to the shorter loci from merged reads. We initially analysed the two data sets separately to assess consistency of phylogenetic and distance-based signals before combining them. Complicating this, our samples came from plants with differing ploidy levels, increasing the number of alleles that might be missed by the low coverage sequencing. While ploidy could be a considerable problem in our system (with both diploid and tetraploid populations in the same species), recent work [71] suggests it is a minor concern for these data sets and is largely addressed by ploidy filters such as we applied through PyRAD. Our study focused on detecting genomic divergence and phylogenetic signal (potentially less impacted by missing alleles within an individual), but other researchers may be interested in population genetics questions that require more accuracy in detecting all alleles for a given locus. Due to the low coverage, GBS may not be ideal for accurate detection of heterozygotes (see [30]), needed for generating population genetics summary statistics, and in non-model systems GBS data sets might be better substituted with an approach with greater sequencing depth, such as RAD sequencing [16].

The missing data seem to have particularly affected our use of SNAPP. Because SNAPP requires that a SNP be present in each terminal taxon and we were analysing eleven taxa, the data set of available SNPs was massively decreased, possibly contributing to the poor resolution observed. The poor resolution may also be due to the limitation to a single SNP per locus, a limitation shared by SVDquartets, which also produced largely unresolved trees. It may be that while the analyses that can use multiple parsimony informative sites from each locus (e.g. RAxML, ASTRAL) can recover strong genomic signal, the restriction to a single SNP per locus erodes signal and SNAPP is unable to resolve relationships, especially in areas of the tree with short branch lengths characteristic of rapid divergence. SNAPP has been used successfully with GBS (e.g. [12]), so it may also be that idiosyncracies of relationships in the *T*. *basedowii* complex contributed to the lack of resolution (i.e. real biological incongruence). For GBS data sets with a large amount of missing data and study systems with recently diverged species, we recommend including analyses that can use multiple SNPs per locus as an alternative to SNAPP.

#### Optimising assembly parameters

When first approaching bioinformatic assembly of loci, it is not always clear what value is most appropriate for a given parameter in a software program like PyRAD or Stacks. We found that the assembly parameter optimisation approach based on Mastretta-Yanes et al. [25] was useful in helping to select reasonable parameter values and get a sense of how our data were impacted by each parameter. The inherent trade-offs between the number of loci and SNPs recovered and error rates and genetic distances made deciding on optimal parameters subjective, and researchers should explore the impact of assembly parameters to tailor the selection for their purposes (e.g. minimising error rates may be more important for a population genetic study). In our study, assembly parameters (particularly clustering threshold and minimum sample coverage) impacted downstream phylogenetic and species tree analyses, especially concatenation approaches. Our preliminary RAxML runs across a broad range of parameter values (S1 and S2 Figs) clearly show that there are conflicting signals in the genomic data sets for different values. We recommend selecting an optimal parameter set following the approach of [25], but also exploring how key parameters impact phylogenetic inference to get a better sense of uncertainty in the data set.

#### Phylogenetic signal and conflict

Bioinformatic assembly of GBS loci may produce data sets with conflicting phylogenetic signals. We specifically looked for phylogenetic conflict across assembly parameters to evaluate how various analyses handled it. For the combined data, RAxML analyses produced well-supported but conflicting topologies depending on the amount of missing data and loci, while TNT analyses better reflected (indicated less support) the uncertainty in the tree recovered for the configuration A data set, though they still recovered conflicting topologies between data sets. In contrast, while the species tree analyses differed in their support values between data sets, they produced largely congruent trees. This difference between methods might reflect the better performance of coalescent approaches that account for gene tree conflict in data sets with large amounts of incomplete lineage sorting (likely in a group of closely related species) compared to concatenation [72,73]. While species tree approaches typically require more computational time (partly because gene trees have to be generated for every locus prior to the analysis), we found them valuable to incorporate alongside traditional phylogenetic approaches for comparison, and useable with reduced representation genomic data of decent read lengths.

**Fig 10:**
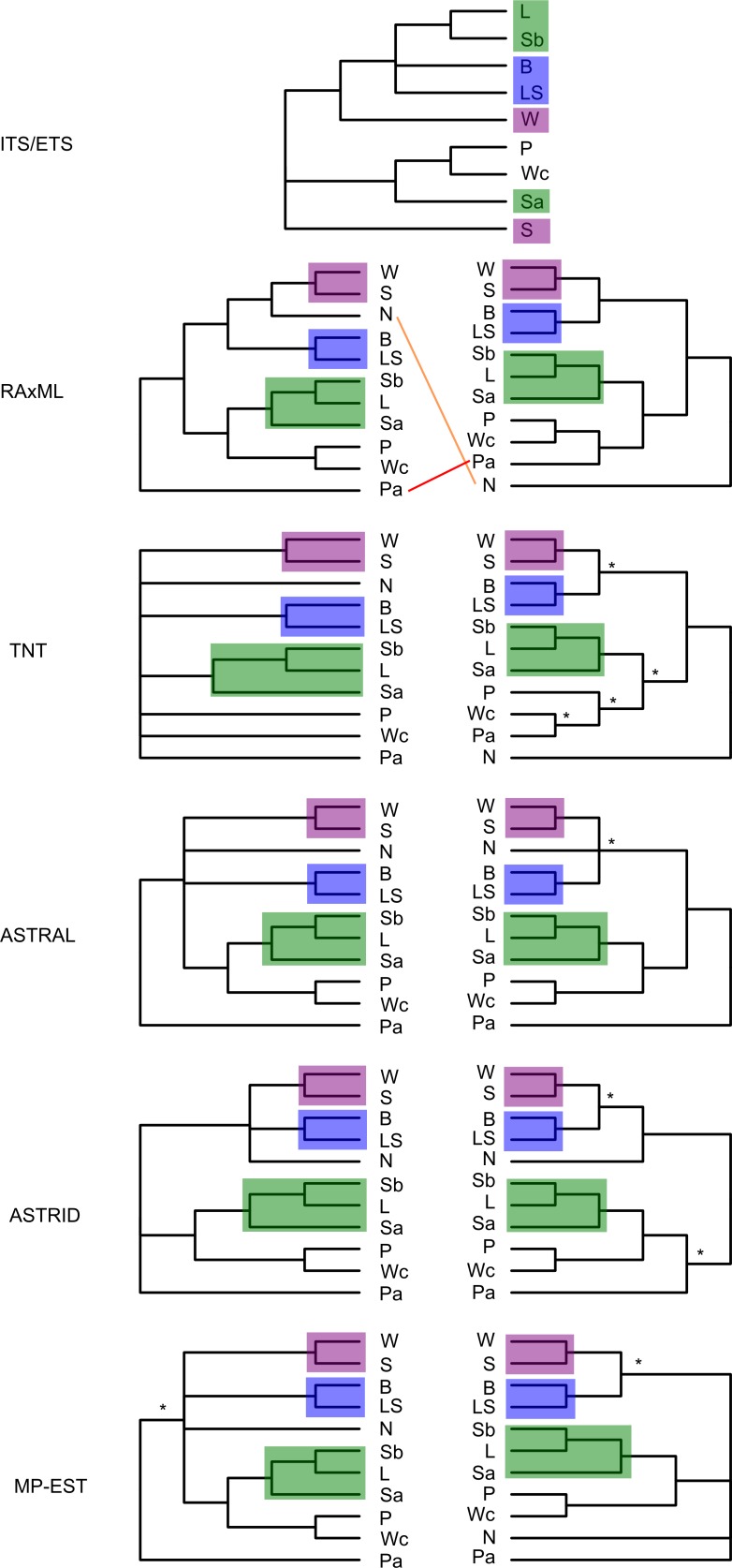
Topologies for the *Triodia basedowii* complex across analyses for two assemblies. 0.88/0.91 (merged/unmerged) clustering threshold, 50% minimum taxon coverage (left) and 0.88/0.82 clustering threshold, 25% minimum taxon coverage (right). Branches with less than 80% support are collapsed. Asterisks designate clades not present in the alternate assembly. Clades consistent across analyses are highlighted. B: *T*. *basedowii*, L: *T*. *lanigera*, LS: *T*. "LSandy", N: *T*. "nana", P: *T*. "Peed", Pa: *T*. "Panna", S: *T*. "Shov", Sa: *T*. "shova", Sb: *T*. "shovb", W: *T*. "War", Wc: *T*. "wcoast". ITS/ETS results are from Anderson et al. [46].

While we recovered some degree of resolution for relationships in the complex (Fig 10), the topological conflicts and low support for some nodes in the trees may reflect real biological conflict caused by incomplete lineage sorting and/or hybridization between recently diverged species. Even large genomic data sets may be unable to resolve bifurcating patterns when the real process is substantially reticulate or rapid; in such cases, the lower support reflects valuable biological information about divergence patterns in the study system. Favouring results that give high topological support (e.g. RAxML with concatenated data) is therefore potentially problematic for understanding natural systems, and researchers would benefit from the inclusion of methods that may better reflect real uncertainty (e.g. lower support values in species tree approaches).

Despite differences, the various analyses consistently supported certain clades (highlighted in Fig 10) regardless of assembly parameters or method of analysis, suggesting there is strong genomic signal supporting their distinction. Across all the well-supported topologies, there is a common trend of more resolution for the configuration B data set, which had more missing data and more loci than the configuration A data set. Greater resolution with more missing data (and therefore more loci) has been previously highlighted for large data sets [74]. Applying strict filtering for sample coverage quickly reduces the size and phylogenetic informativeness of GBS data sets, so researchers are encouraged to consider low sample coverage thresholds when inferring phylogenies. We also found that setting a strict filtering level for the number of SNPs in a locus (to remove poorly aligned or repetitive regions) removed good loci and phylogenetic signal. While a small number of SNPs per locus is a reasonable filter for studies on one or two species, it can be detrimental to phylogenomic studies with multiple divergent species and outgroups. In our study system, we found that loci in one species might differ from another at a few positions, but differ from a different relative at other positions, often resulting in many SNPs (and phylogenetically informative positions) recovered per locus. As such, researchers working in similarly multi-species systems would benefit from not setting strict limits on the number of SNPs in a final locus.

### The *Triodia basedowii* Species Complex

Our GBS data provide a first measure of genomic variation in the *Triodia basedowii* species complex and new insights into the distinction of taxa and their evolutionary relationships. There is a close correspondence between genomic entities and *a priori* taxon identifications, across large geographical ranges and even where taxa co-occur, consistent with the hypothesis that these taxa are distinct species.

Well-supported clades recovered in phylogenetic and species tree analyses are partly congruent and incongruent with those found in Anderson et al. [46]. In some cases, GBS results provide support where previous results were ambiguous. For example, Anderson et al. [46] hypothesised a sister relationship between *T*. "Shov" and *T*. "War" based on morphology and the presence of shared ITS copies, but found no or ambiguous support for this in molecular phylogenies using ITS/ETS and a chloroplast marker. Our GBS analysis strongly supports this sister relationship, with phylogenetic and species trees always recovering it and clustering analyses showing the two entities to be close but distinct. Similarly, Anderson et al. [46] were unable to resolve *T*. "LSandy" as distinct from *T*. *basedowii* despite morphology and field observations suggesting that it is. In our GBS trees, *T*. "LSandy" and *T*. *basedowii* were consistently recovered as sisters, with clustering analyses and branch lengths in concatenation and MP-EST trees suggesting substantial divergence between them. Anderson et al. [46] also found insufficient evidence to recognize the putative taxon *T*. "shovb" as distinct from *T*. *lanigera*, and the ITS/ETS analysis placed the combined taxon as part of a polytomy with *T*. *basedowii*. Our GBS results consistently recover their close relationship and limited divergence, supporting the former conclusion (we continue to treat them as divergent populations of a single species), but place them as sister to *T*. "shova" rather than *T*. *basedowii*. This agrees with chloroplast data [46], suggesting that the ITS result may represent introgression from *T*. *basedowii*.

The new putative taxa *T*. "nana" and *T*. "Panna" were consistently distinct across analyses but unstably placed in the GBS trees. Branch lengths subtending these two taxa are consistently long (especially for *T*. "Panna"), suggesting that these morphologically distinct entities should be recognised as distinct new species in the complex. *T*. "Panna" is of particular importance as it is known from a single population, and, despite being a tetraploid, has the lowest heterozygosity for taxa in our study based on statistics from the PyRAD assembly. This suggests that the population has experienced a severe bottleneck, which together with its restricted distribution makes it of conservation concern.

Ploidy variation in the *Triodia basedowii* complex does not follow taxonomic boundaries, suggesting that most polyploid events represent sporadic cases of autopolyploidy arising recently within species. Our GBS analyses show that the widespread *T*. *basedowii* forms a distinct genomic cluster, with some distinction between populations but without tetraploid populations clustering distinctly from diploid populations (e.g. central Australian populations cluster together despite different ploidy levels). Multiple ploidy levels are also found in *T*. *lanigera* (including some of its *T*. "shovb" populations) and in a population of putative hybrids between tetraploid *T*. *lanigera* and diploid *T*. "shova", suggesting that differing ploidy is not acting as a strong reproductive barrier in this case. The co-occurrence of individuals with different ploidy (as in e.g. a population of *T*. "wcoast") is rare in the complex, however, suggesting some segregation between ploidy levels may occur. Outside the complex, indirect evidence in the form of heterozygosity statistics for PyRAD loci (elevated in tetraploids in the unmerged data) suggest there may be diploid plants in the tetraploid *T*. *concinna* population. These results suggest that polyploidy has not been a major speciation driver in the complex.

Our species tree analyses are helpful in assessing the distinction of entities that are dispersed or not sister in the trees (e.g. *T*. "Shov" from *T*. *basedowii*), but because we designated taxa *a priori*, these analyses do not help to assess whether sisters are distinct (e.g. *T*. "Peed" from *T*. "wcoast"). Our decision to recognise taxa in these cases depends on assessing the degree of genomic divergence (from clustering analyses and phylogenetic branch lengths) along with supporting evidence (from e.g. morphology or distribution). For example, the geographically disjunct putative taxa *T*. "Peed" (diploid) and *T*. "wcoast" (tetraploid/diploid) have some morphological distinction but are indistinguishable in ITS/ETS [46]. Our GBS results suggest moderate divergence between the two entities, and they do not always form a single clade (e.g. paraphyletic in Fig 4B, S13B and S14B Figs), but our sampling of *T*. "wcoast" is relatively poor. With these ambiguous results, we conservatively treat them as divergent populations of one species, pending more extensive sampling. This example contrasts with that of *T*. *basedowii* and *T*. "LSandy", where there is more clearly morphological distinction, a more substantial degree of genomic divergence, and co-occurrence without evident mixing.

Localities where putative taxa co-occur provide a useful test for species distinction and an opportunity to assess the frequency of hybridization. Anderson et al. [46] provided evidence of hybridization between some taxa in the *T*. *basedowii* complex, and our GBS results both support and contradict that evidence. GBS results from one population ("Amb") support previous evidence for hybridization between *T*. *lanigera* and *T*. "shova". Individuals with intermediate morphologies clearly form genomic clusters between the putative parental species in the PCoA (Fig 5B), with those identified as more like *T*. "shova" clustering closer to that taxon and similarly for *T*. *lanigera*. In contrast, a population ("mixSPH") containing both *T*. *lanigera* and *T*. "shova" that showed evidence of some ITS copy sharing (Table 6) did not show evidence of genomic mixing in our results. Multiple *T*. "shova" individuals possess both *T*. *lanigera* and *T*. "shova" ITS copies, while the same cannot be said for *T*. *lanigera* individuals, and our GBS results place all samples from this population with their respective taxa and without suggestions of genomic intermediacy.

**Table 6:** ITS copy types for co-occurring *Triodia lanigera* and *T*. "shova".

Sample name	Taxon	ITS copy type	GenBank reference	Note
T_lan_Barrett_4107_GillamCk	*T*. *lanigera*	A	KU173242	*T*. *lanigera* reference
T_lan_Anderson_16_SPortHedland	*T*. *lanigera*	A		pop. voucher
T_lan_Anderson16_mixLSPH_2	*T*. *lanigera*	A		
T_lan_Anderson16_mixLSPH_6	*T*. *lanigera*	A		
T_lan_Anderson16_mixLSPH_8	*T*. *lanigera*	A		
T_lan_Anderson16_mixLSPH_10	*T*. *lanigera*	A		
T_lan_Anderson16_mixLSPH_14	*T*. *lanigera*	A		
T_shova_Barrett_4106_GillamCk	*T*. "shova"	B	KU173287	*T*. "shova" reference
T_shova_Anderson15_mixSPH_3	*T*. "shova"	B		
T_shova_Anderson15_mixSPH_7	*T*. "shova"	B		
T_shova_Anderson15_mixSPH_9	*T*. "shova"	B		
T_shova_Anderson15_mixSPH_17	*T*. "shova"	B		
T_shova_Anderson_15_SPortHedland	*T*. "shova"	A + B		pop. voucher
T_shova_Anderson15_mixSPH_11	*T*. "shova"	A + B		
T_shova_Anderson15_mixSPH_15	*T*. "shova"	A + B		
T_shova_Anderson_19_TwoCamelCk	*T*. *lanigera* x "shova"	A + B	KU173284, KU173285	hybrid reference

Sequences sometimes had heterozygous positions so that they differed slightly from the references, but were still identifiably either of the two copy types. Samples with "SPortHedland", "mixLSPH" or "mixSPH" in their names are from the "mixSPH" mixed population.

The contrasting results suggest that there is limited introgression of rDNA despite relatively strong barriers across the rest of the genome. Similarly, a population ("RoyH") of individuals found to possess ITS copies characteristic of *T*. "Shov" and *T*. "War" showed no evidence of genomic mixing in our results following resampling. Samples from the population clustered with *T*. "Shov", suggesting that introgression may be restricted to rDNA or that our second sampling for GBS (only four plants) comprised parental plants rather than hybrids, if the population contained both. In other populations with co-occurring taxa (e.g. "Roy1" comprising *T*. "Shov" and *T*. *basedowii*, with one probable misidentification, and "Nifty" comprising *T*. "War" and *T*. *basedowii*) there is no evidence of genomic mixing or rDNA introgression, which suggests these taxa are reproductively isolated.

## Conclusion

By addressing GBS bioinformatic challenges, such as overlapping reads following imperfect size selection and paired-end read locus duplication, a biologically meaningful phylogenetic signal from across the genome can be extracted for study systems lacking prior genomic knowledge. The continuing improvement in next-generation sequencing read length holds promise for using analytical methods that rely on extracting phylogenetic signal from each locus, such as new species tree approaches. Here we have demonstrated their use with our assembled GBS loci and shown that they are more robust to variation in assembly parameters than commonly used concatenation approaches. Our GBS data and analyses have improved the resolution of relationships in the *T*. *basedowii* complex and provided new insights into processes influencing evolution of the group (e.g. polyploidy and partial introgression). These results will support an upcoming taxonomic revision of the complex, with the recognition of new species including one of conservation significance, and complement the findings of Anderson et al. [46] with regard to the high diversity of the complex in the Pilbara region of Western Australia. We encourage other researchers working in similarly difficult taxonomic systems to use the relatively affordable and accessible GBS approach outlined here for generating and analysing genomic data.

## Supporting Information

S1 File Sample information for sequencing and analyses. (XLS)

S2 File Details of GBS Library Prepartion. (XLS)

S3 File Select scripts used in this study. (ZIP)

S1 Fig RAxML trees for the merged data set. Trees are shown across three clustering thresholds (0.82, 0.88, 0.97) and three minimum taxon coverage levels (25%, 50%, 75%). Support values from 100 bootstrap replicates are only shown for branches with <100% support. Scale bar units are branch lengths from RAxML. Two selected trees are boxed (solid for the selected coverage level; dashed for a well-supported conflicting topology). B: *T*. *basedowii*, C: *T*. *concinna*, L: *T*. *lanigera*, LS: *T*. "LSandy", N: *T*. "nana", O: additional outgroups (*T*. *wiseana*, *T*. *intermedia*), P: *T*. "Peed", Pa: *T*. "Panna", Pl: *T*. *plurinervata*, S: *T*. "Shov", Sa: *T*. "shova", Sb: *T*. "shovb", W: *T*. "War", Wc: *T*. "wcoast". *Clade only includes some samples of this taxon.
(TIF)

S2 Fig RAxML trees for the unmerged data set. Trees are shown across three clustering thresholds (0.82, 0.91, 0.97) and three minimum taxon coverage levels (25%, 50%, 75%). Support values from 100 bootstrap replicates are only shown for branches with <100% support. Scale bar units are branch lengths from RAxML. Two selected trees are boxed (solid for the selected coverage level; dashed for a well-supported conflicting topology). B: *T*. *basedowii*, C: *T*. *concinna*, L: *T*. *lanigera*, LS: *T*. "LSandy", N: *T*. "nana", O: additional outgroups (*T*. *wiseana*, *T*. *intermedia*), P: *T*. "Peed", Pa: *T*. "Panna", Pl: *T*. *plurinervata*, S: *T*. "Shov", Sa: *T*. "shova", Sb: *T*. "shovb", W: *T*. "War", Wc: *T*. "wcoast".
(TIF)

S3 Fig Recovered loci/SNPs and error rates for the PyRAD parameter optimisation. Merged data are designated with circles, unmerged with triangles. The replicate of *T*. *basedowii* is designated with hollow points, the replicate of *T*. "shova" with solid points. Parameters are c: clustering threshold; d: minimum depth for a statistical base call; h: maximum number of shared heterozygous positions; mr: minimum read depth for a dereplicate; n: maximum number of low quality sites; pl: maximum number of alleles per locus. Parameter values that were selected are boxed.
(TIF)

S4 Fig Intra-population genetic distances for samples from *T*. *basedowii* population "Andado" across PyRAD parameter values. (A) merged, and (B) unmerged data sets. Parameters are c: clustering threshold; d: minimum depth for a statistical base call; h: maximum number of shared heterozygous positions; mr: minimum read depth for a dereplicate; n: maximum number of low quality sites; pl: maximum number of alleles per locus. Parameter values that were selected are boxed.
(TIF)

S5 Fig Intra-population distances across 10 populations for parameter c. (A) merged and (B) unmerged data sets.
(TIF)

S6 Fig Intra-population distances across 10 populations for parameter d. (A) merged and (B) unmerged data sets.
(TIF)

S7 Fig Intra-population distances across 10 populations for parameter h. (A) merged and (B) unmerged data sets.
(TIF)

S8 Fig Intra-population distances across 10 populations for parameter mr. (A) merged and (B) unmerged data sets.
(TIF)

S9 Fig Intra-population distances across 10 populations for parameter n. (A) merged and (B) unmerged data sets.
(TIF)

S10 Fig Intra-population distances across 10 populations for parameter pl. (A) merged and (B) unmerged data sets.
(TIF)

S11 Fig SNAPP trees and cloudograms for the PyRAD-selected unlinked SNPs and two assemblies. (A) 0.88/0.91 (merged/unmerged) clustering threshold, (B) 0.88/0.82 clustering threshold. Node values are posterior probabilities. Scale bars are coalescent units.
(TIF)

S12 Fig SNAPP trees and cloudograms for the biased unlinked SNPs and two assemblies. (A) 0.88/0.91 (merged/unmerged) clustering threshold, (B) 0.88/0.82 clustering threshold. Node values are posterior probabilities. Scale bars are coalescent units.
(TIF)

S13 Fig SVDquartets trees for the PyRAD-selected unlinked SNPs and two assemblies. (A) 0.88/0.91 (merged/unmerged) clustering threshold, (B) 0.88/0.82 clustering threshold. Species trees (left) and lineage trees (right). Support from 100 bootstrap replicates are shown for branches with support >75%. Branch lengths do not reflect divergence.
(TIF)

S14 Fig SVDquartets trees for the biased unlinked SNPs and two assemblies. (A) 0.88/0.91 (merged/unmerged) clustering threshold, (B) 0.88/0.82 clustering threshold. Species trees (left) and lineage trees (right). Support from 100 bootstrap replicates are shown for branches with support >75%. Branch lengths do not reflect divergence.
(TIF)
