# Supplementary figures and images for: Genotyping-by-Sequencing in a Species Complex of Australian Hummock Grasses (Triodia): Methodological Insights and Phylogenetic Resolution
Source: PLoS One. 2017 Jan 30;12(1):e0171053. doi: 10.1371/journal.pone.0171053 (PMC5279811; doi:10.1371/journal.pone.0171053)

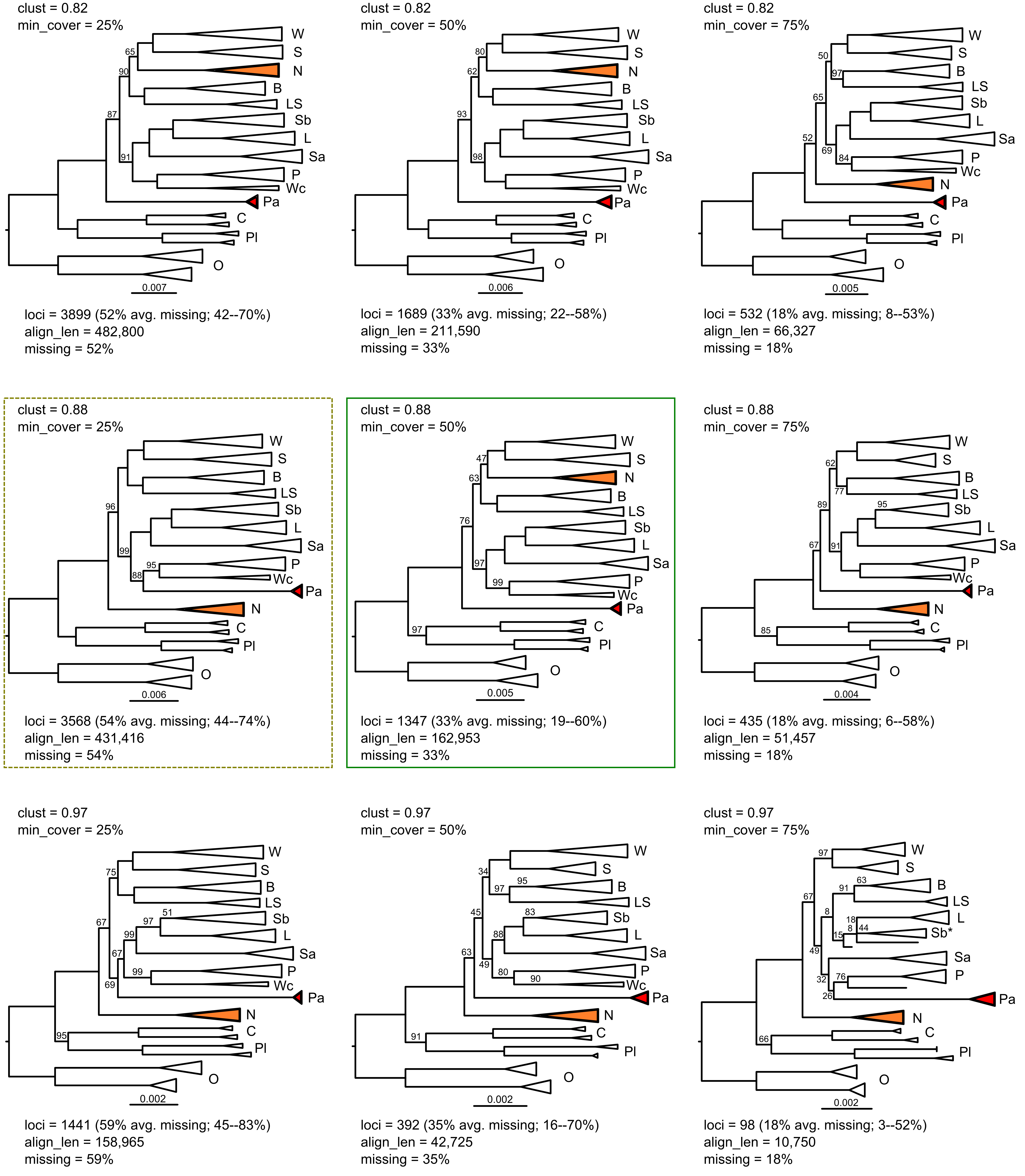

Supplement: S1 Fig — Trees are shown across three clustering thresholds (0.82, 0.88, 0.97) and three minimum taxon coverage levels (25%, 50%, 75%). Support values from 100 bootstrap replicates are only shown for branches with <100% support. Scale bar units are branch lengths from RAxML. Two selected trees are boxed (solid for the selected coverage level; dashed for a well-supported conflicting topology). B: T. basedowii, C: T. concinna, L: T. lanigera, LS: T. "LSandy", N: T. "nana", O: additional outgroups (T. wiseana, T. intermedia), P: T. "Peed", Pa: T. "Panna", Pl: T. plurinervata, S: T. "Shov", Sa: T. "shova", Sb: T. "shovb", W: T. "War", Wc: T. "wcoast". *Clade only includes some samples of this taxon. (TIF) [file pone.0171053.s004.tif]

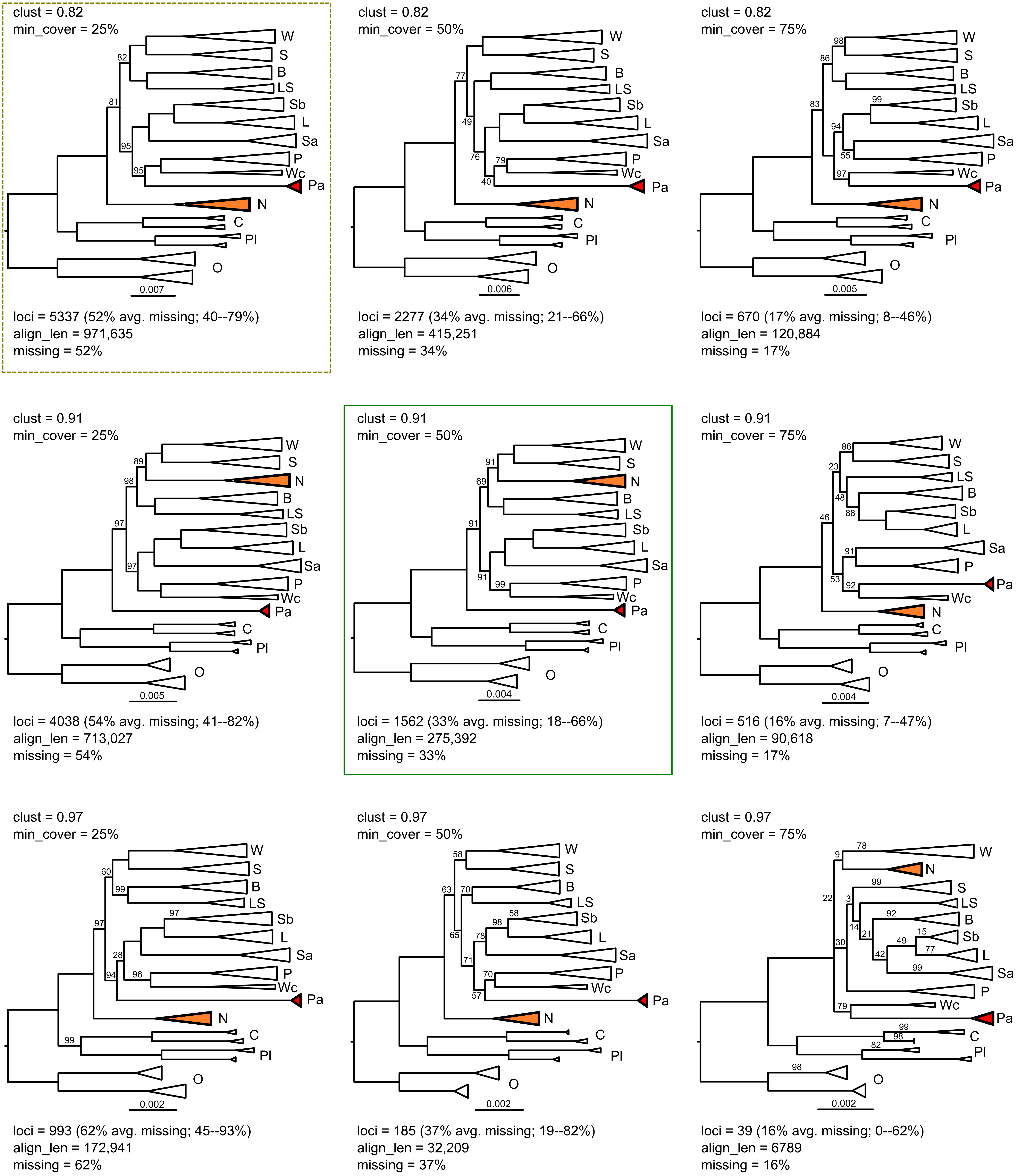

Supplement: S2 Fig — Trees are shown across three clustering thresholds (0.82, 0.91, 0.97) and three minimum taxon coverage levels (25%, 50%, 75%). Support values from 100 bootstrap replicates are only shown for branches with <100% support. Scale bar units are branch lengths from RAxML. Two selected trees are boxed (solid for the selected coverage level; dashed for a well-supported conflicting topology). B: T. basedowii, C: T. concinna, L: T. lanigera, LS: T. "LSandy", N: T. "nana", O: additional outgroups (T. wiseana, T. intermedia), P: T. "Peed", Pa: T. "Panna", Pl: T. plurinervata, S: T. "Shov", Sa: T. "shova", Sb: T. "shovb", W: T. "War", Wc: T. "wcoast". (TIF) [file pone.0171053.s005.tif]

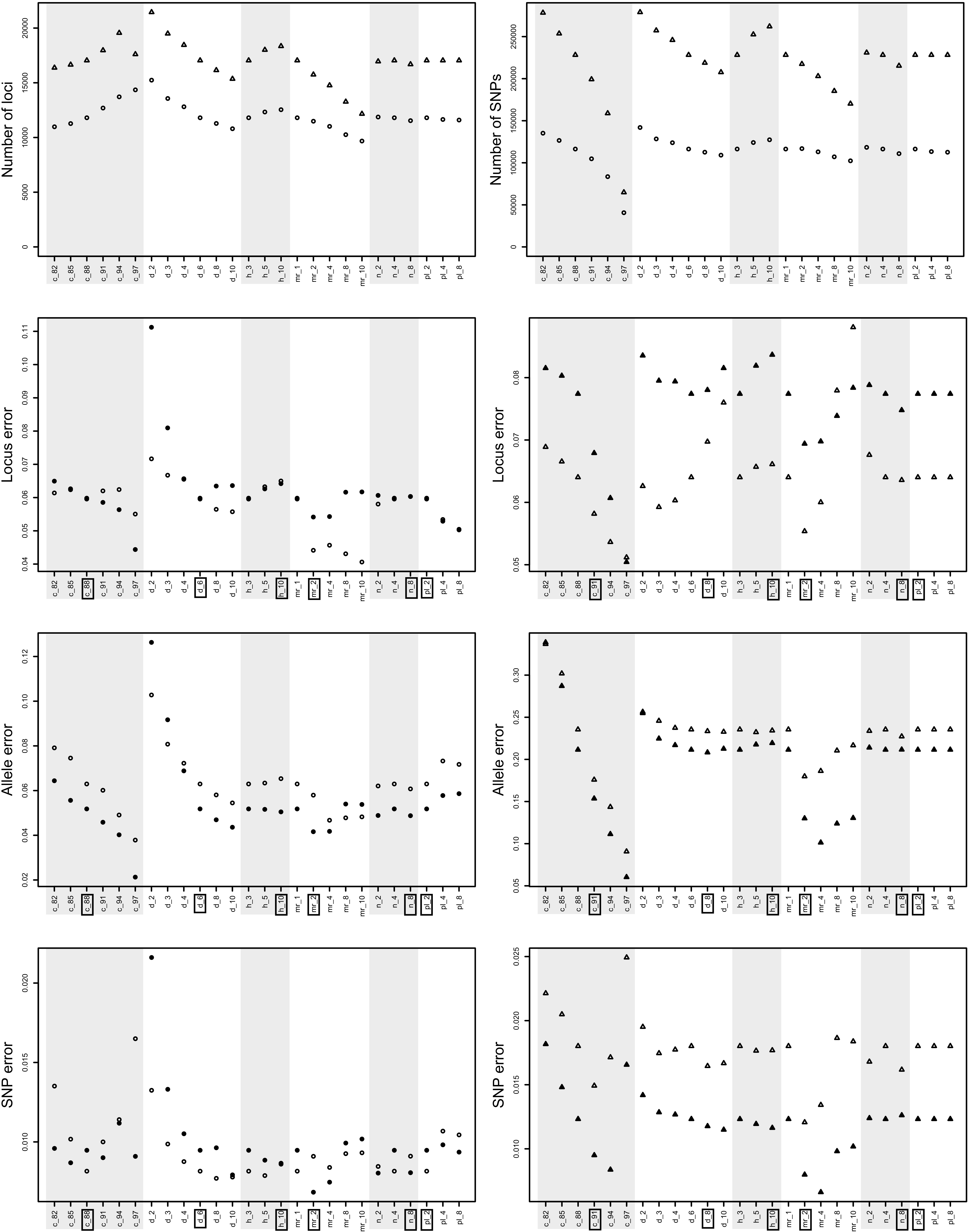

Supplement: S3 Fig — Merged data are designated with circles, unmerged with triangles. The replicate of T. basedowii is designated with hollow points, the replicate of T. "shova" with solid points. Parameters are c: clustering threshold; d: minimum depth for a statistical base call; h: maximum number of shared heterozygous positions; mr: minimum read depth for a dereplicate; n: maximum number of low quality sites; pl: maximum number of alleles per locus. Parameter values that were selected are boxed. (TIF) [file pone.0171053.s006.tif]

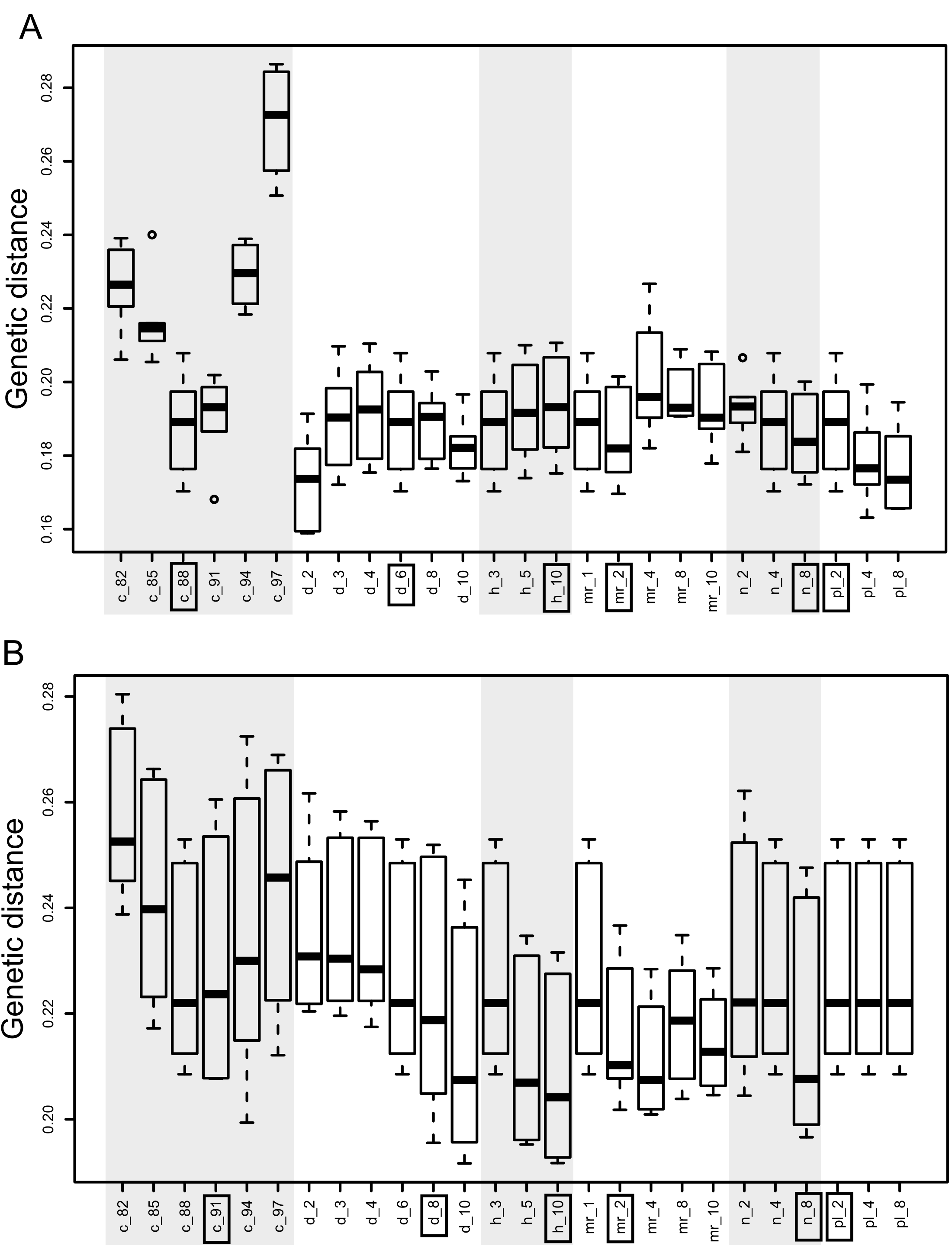

Supplement: S4 Fig — (A) merged, and (B) unmerged data sets. Parameters are c: clustering threshold; d: minimum depth for a statistical base call; h: maximum number of shared heterozygous positions; mr: minimum read depth for a dereplicate; n: maximum number of low quality sites; pl: maximum number of alleles per locus. Parameter values that were selected are boxed. (TIF) [file pone.0171053.s007.tif]

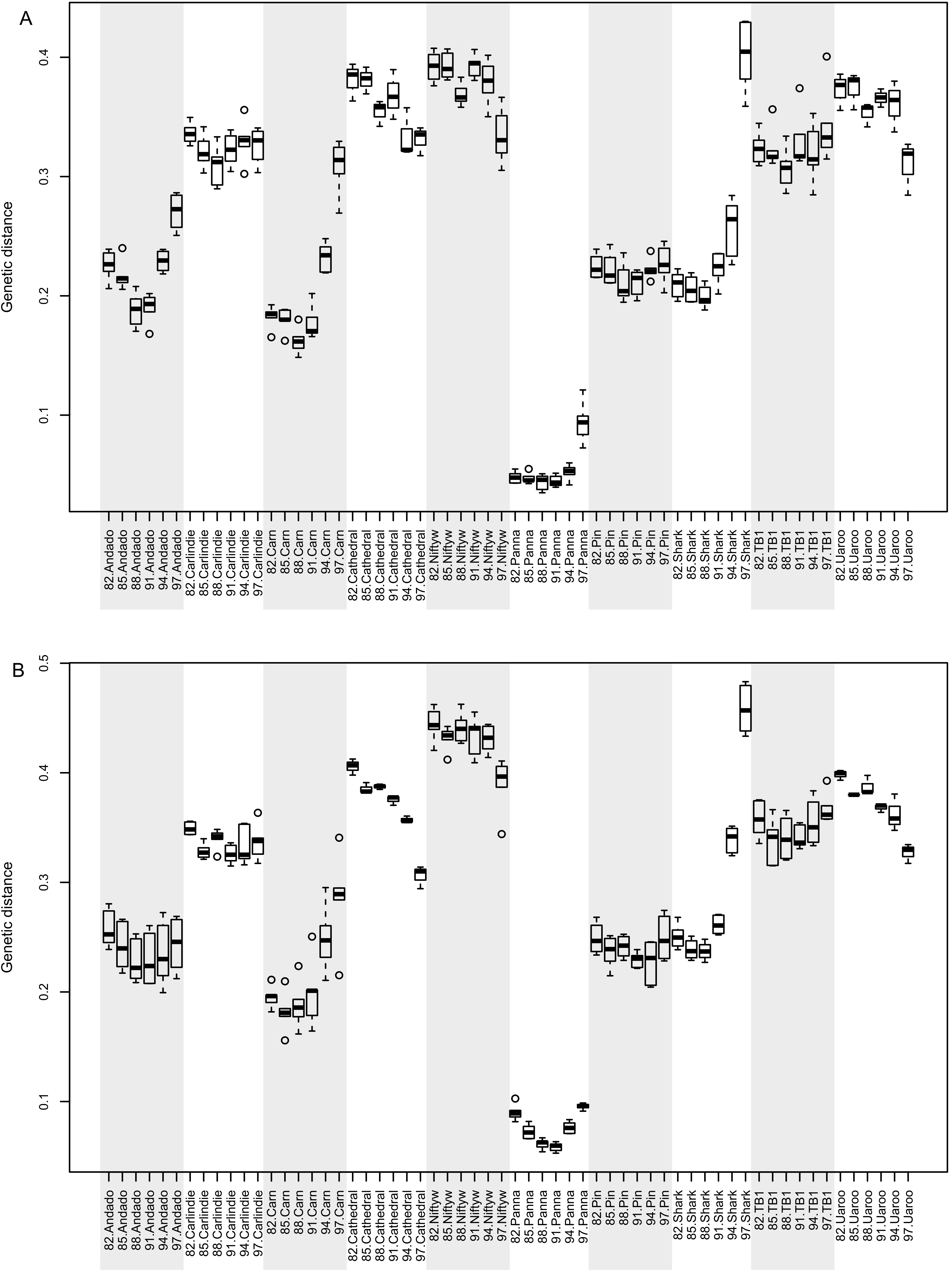

Supplement: S5 Fig — (A) merged and (B) unmerged data sets. (TIF) [file pone.0171053.s008.tif]

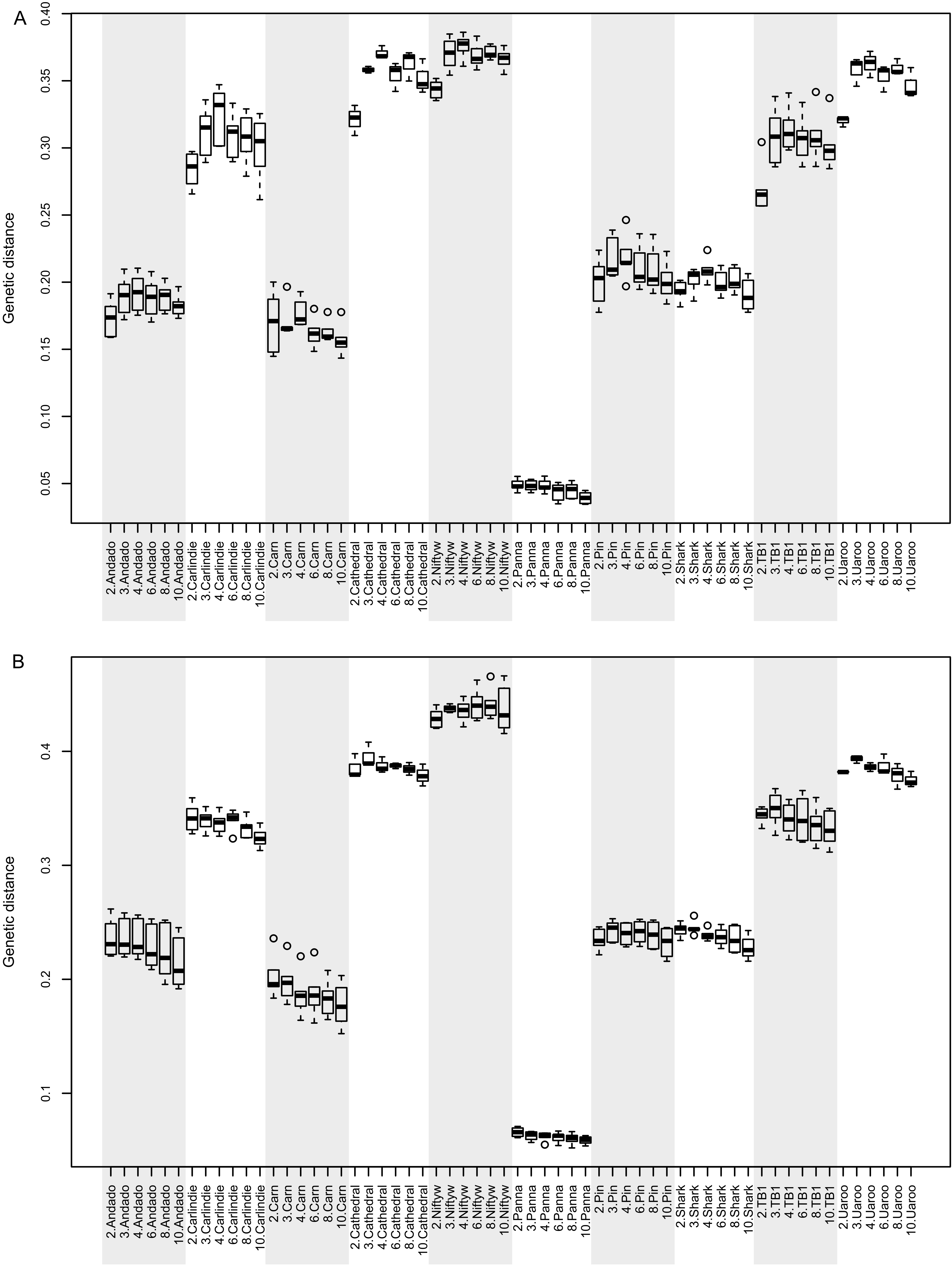

Supplement: S6 Fig — (A) merged and (B) unmerged data sets. (TIF) [file pone.0171053.s009.tif]

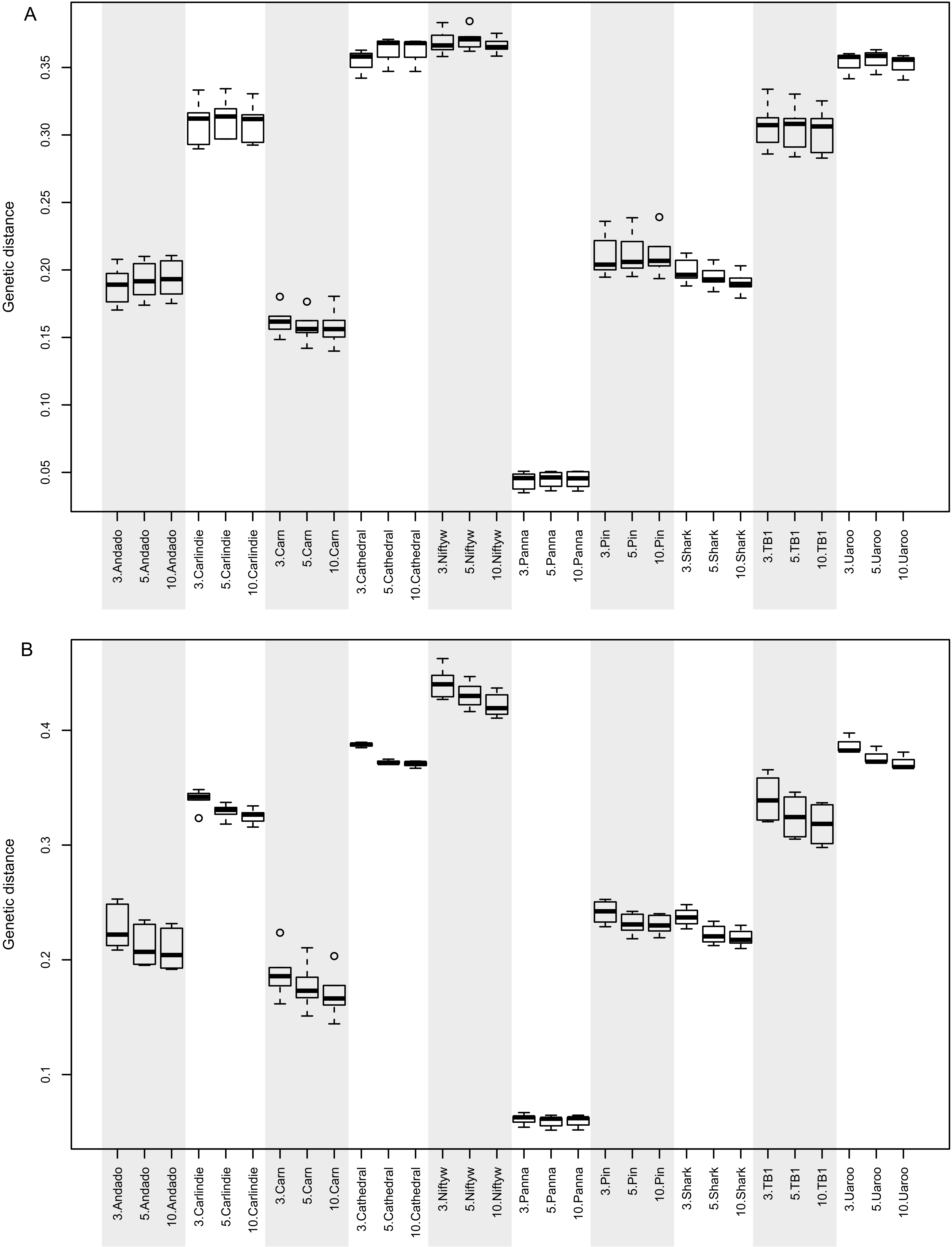

Supplement: S7 Fig — (A) merged and (B) unmerged data sets. (TIF) [file pone.0171053.s010.tif]

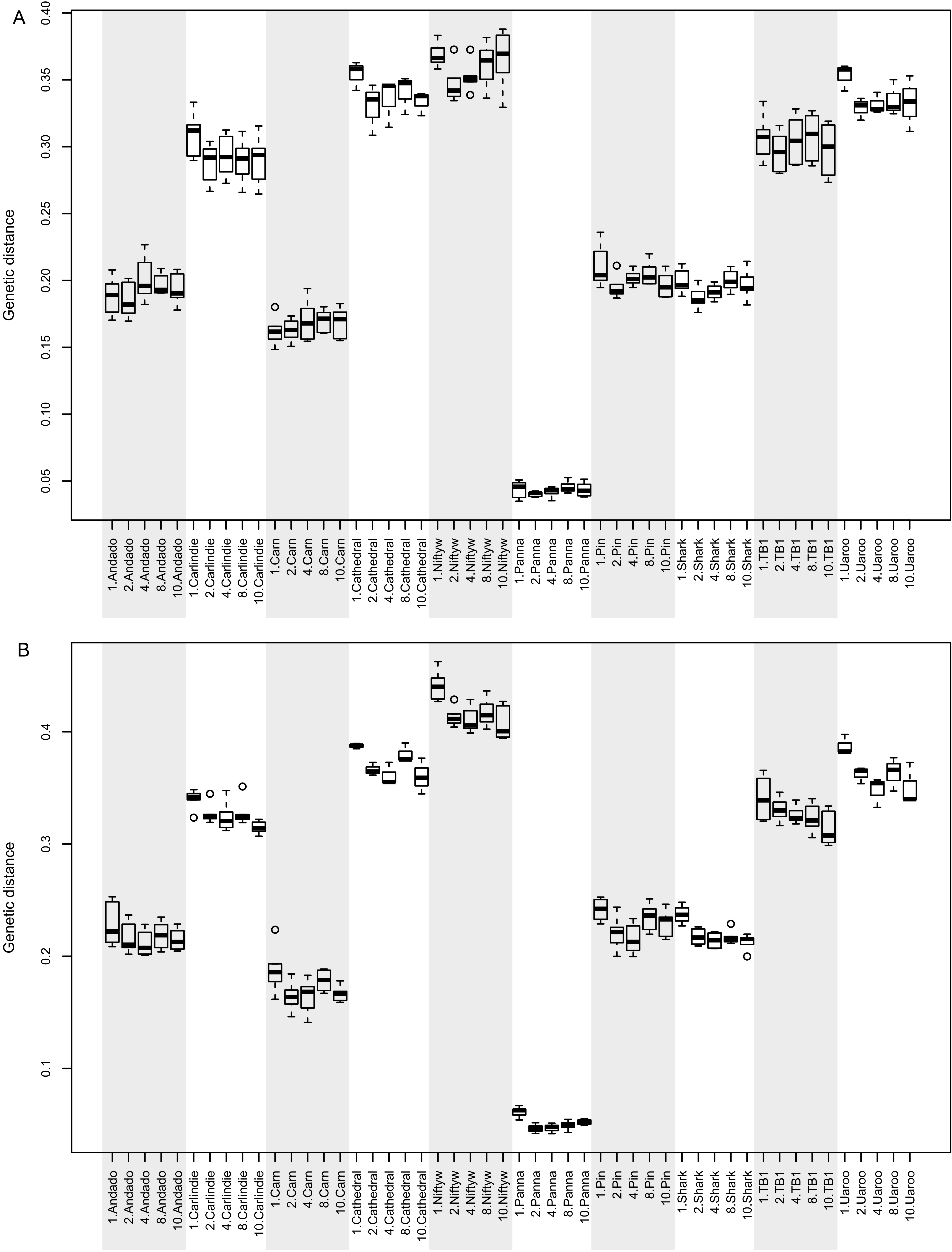

Supplement: S8 Fig — (A) merged and (B) unmerged data sets. (TIF) [file pone.0171053.s011.tif]

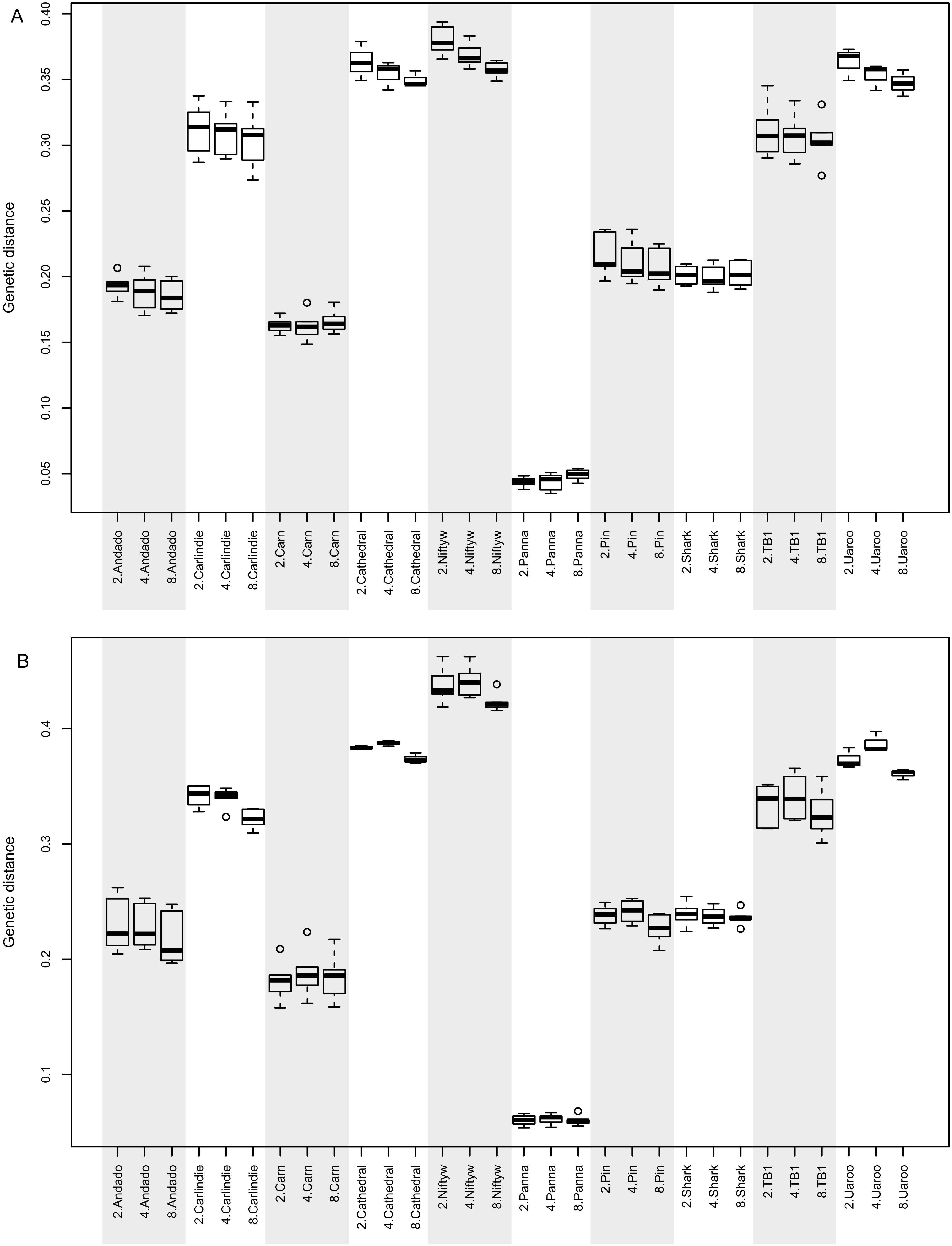

Supplement: S9 Fig — (A) merged and (B) unmerged data sets. (TIF) [file pone.0171053.s012.tif]

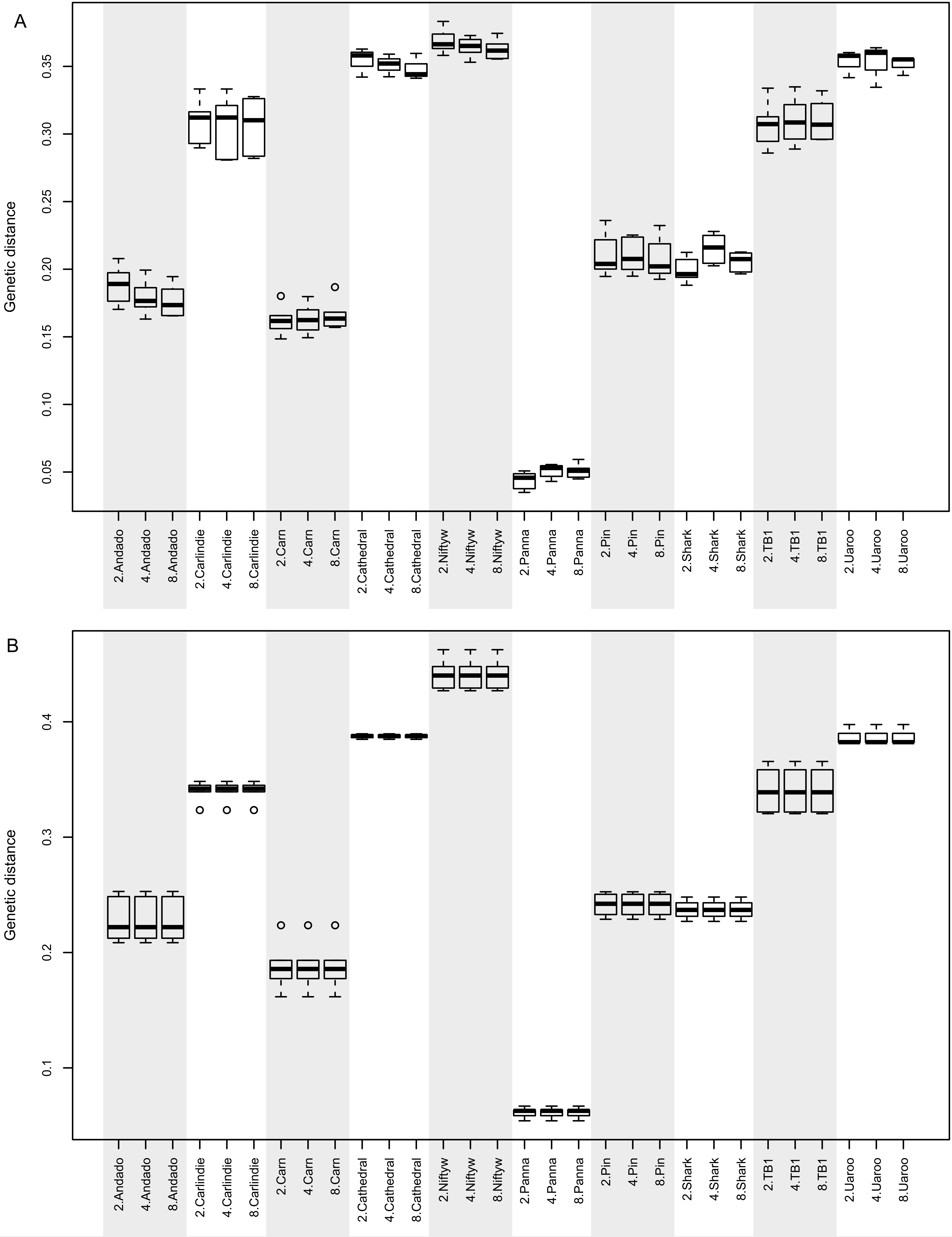

Supplement: S10 Fig — (A) merged and (B) unmerged data sets. (TIF) [file pone.0171053.s013.tif]

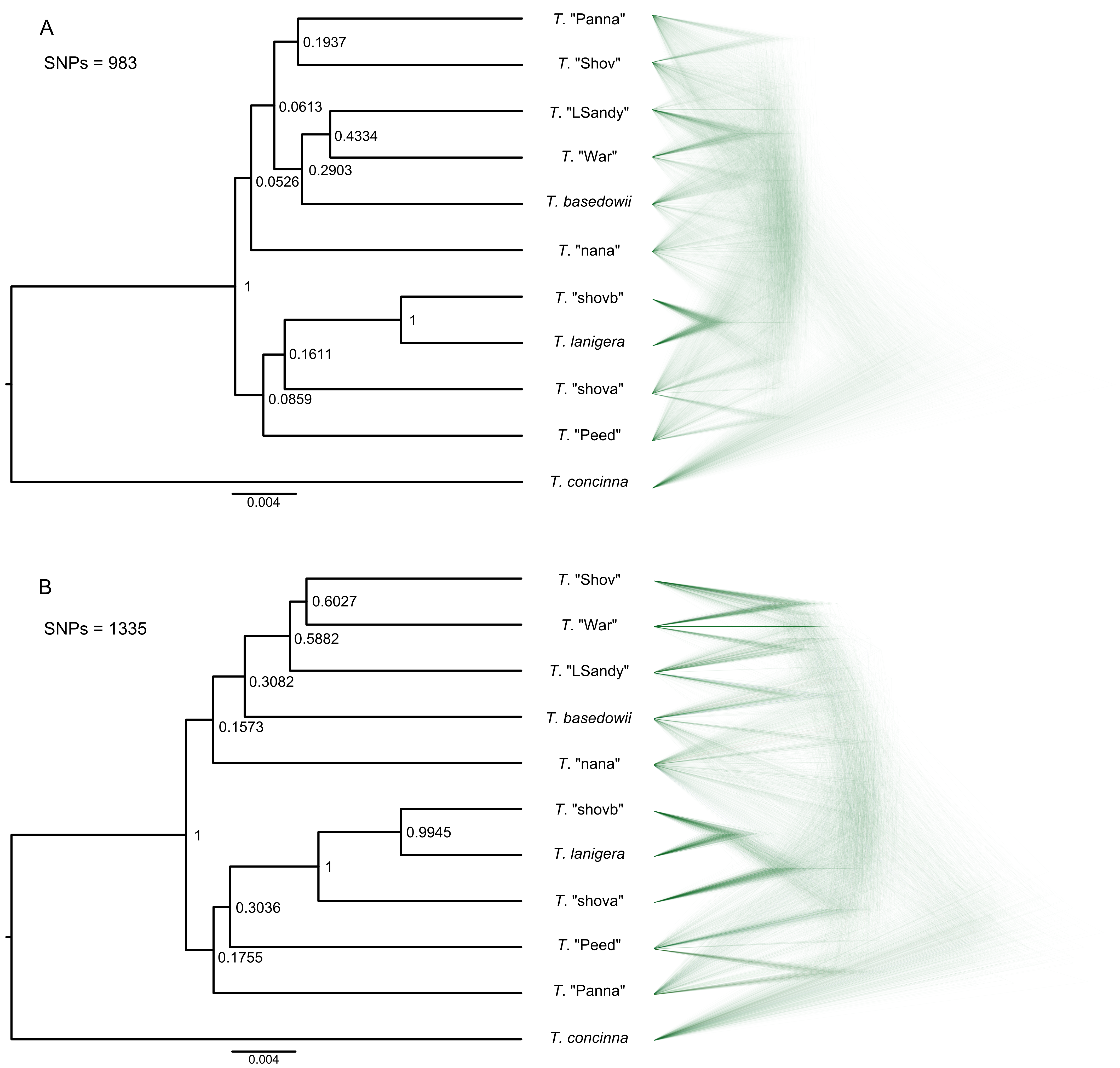

Supplement: S11 Fig — (A) 0.88/0.91 (merged/unmerged) clustering threshold, (B) 0.88/0.82 clustering threshold. Node values are posterior probabilities. Scale bars are coalescent units. (TIF) [file pone.0171053.s014.tif]

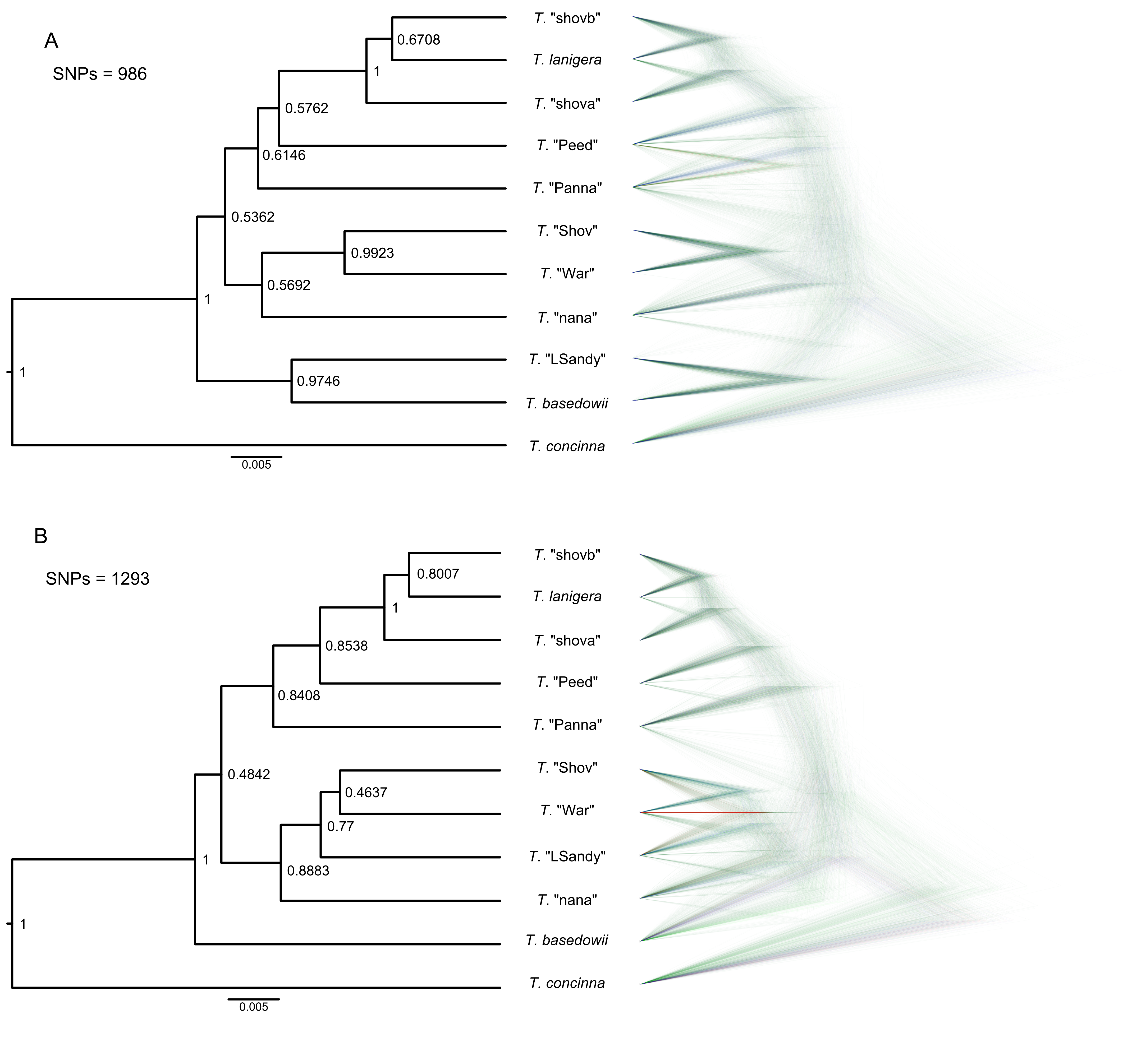

Supplement: S12 Fig — (A) 0.88/0.91 (merged/unmerged) clustering threshold, (B) 0.88/0.82 clustering threshold. Node values are posterior probabilities. Scale bars are coalescent units. (TIF) [file pone.0171053.s015.tif]

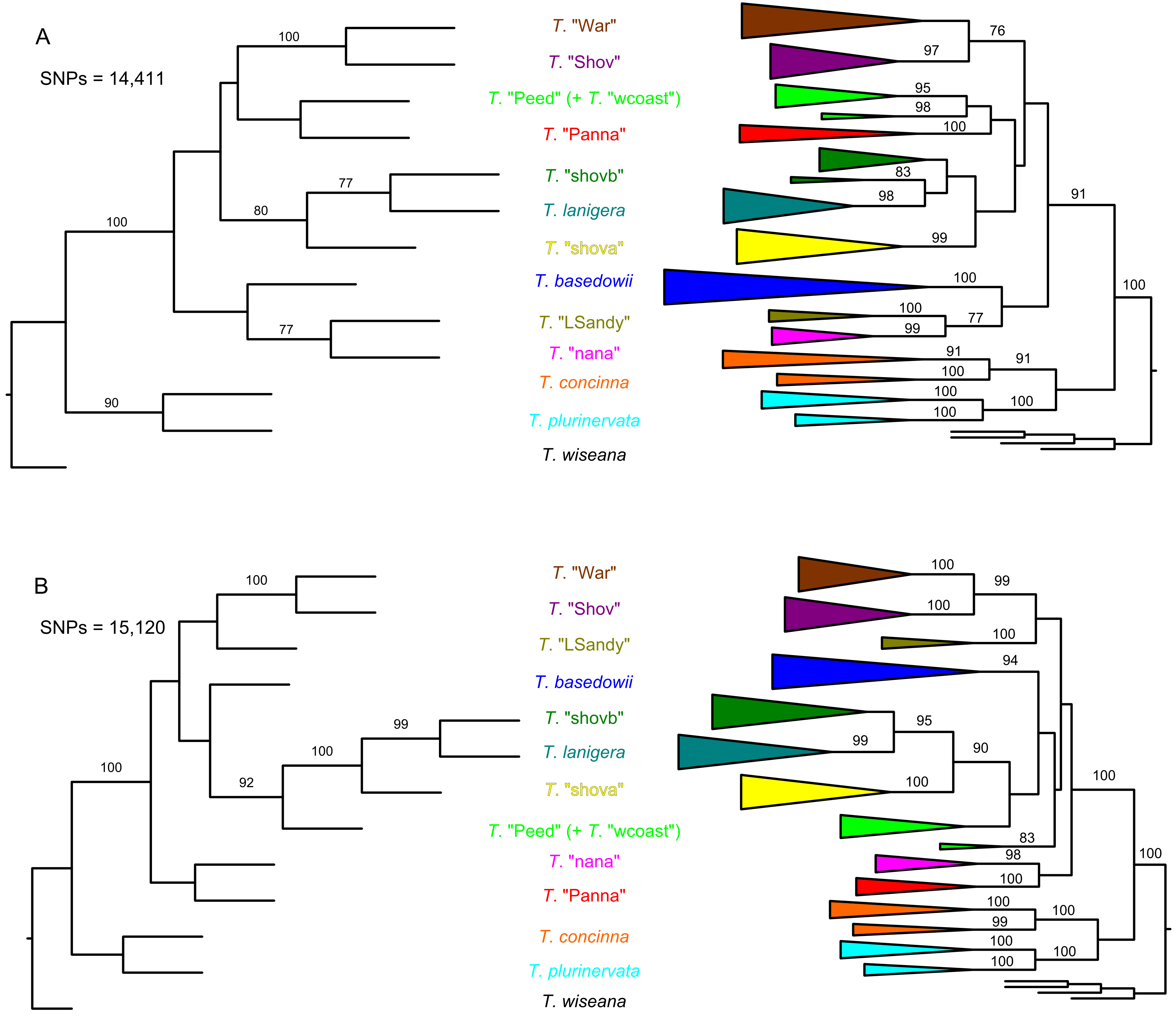

Supplement: S13 Fig — (A) 0.88/0.91 (merged/unmerged) clustering threshold, (B) 0.88/0.82 clustering threshold. Species trees (left) and lineage trees (right). Support from 100 bootstrap replicates are shown for branches with support >75%. Branch lengths do not reflect divergence. (TIF) [file pone.0171053.s016.tif]

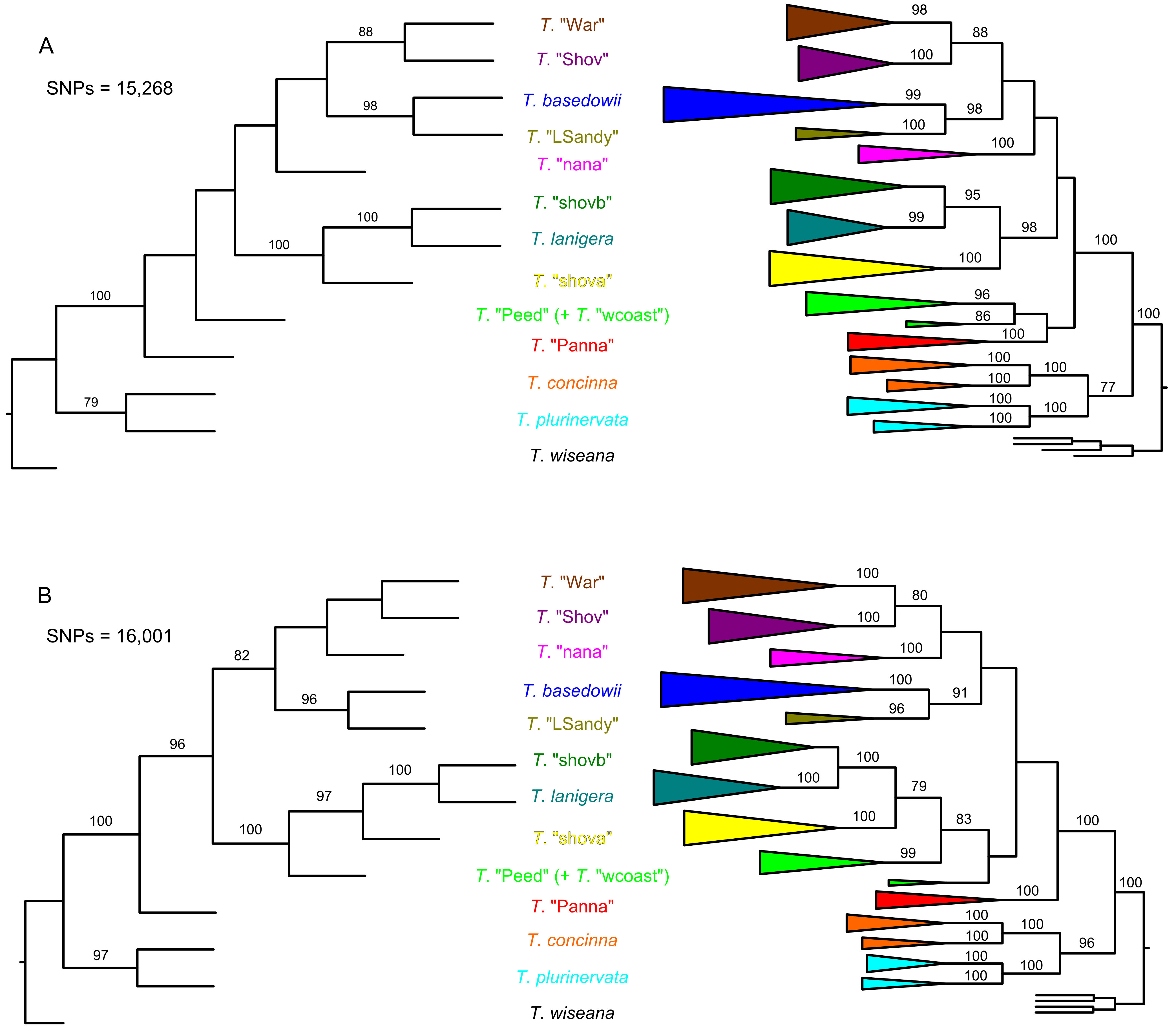

Supplement: S14 Fig — (A) 0.88/0.91 (merged/unmerged) clustering threshold, (B) 0.88/0.82 clustering threshold. Species trees (left) and lineage trees (right). Support from 100 bootstrap replicates are shown for branches with support >75%. Branch lengths do not reflect divergence. (TIF) [file pone.0171053.s017.tif]
